# Supplementary material for: Automated temporalis muscle quantification and growth charts for children through adulthood
Source: Nat Commun. 2023 Nov 9;14:6863. doi: 10.1038/s41467-023-42501-1 (PMC10636102; doi:10.1038/s41467-023-42501-1)
Supplement: Supplementary file 1 — Supplementary Information [file 41467_2023_42501_MOESM1_ESM.pdf]

Supplementary Information for

Automated Temporalis Muscle Quantification and Growth Charts for Children Through Adulthood

INDEX

A.1 PROTOCOL FOR GROUND TRUTH ANNOTATION 3

A.2 HEAD-CIRCUMFERENCE ITMT ADJUSTMENT 5

A.3 FERET DIAMETER 5

A.4 MODEL SENSITIVITY ANALYSIS 5

A.5 MRI QUALITY ASSESSMENT 6

A.6 GAMLSS MODEL DIAGNOSTICS 6

A.7 PRIMARY DATASETS 7

A.8 PHYSICAL BIOMARKERS DEFINITIONS 16

A.9 LEAVE-ONE-STUDY OUT ANALYSIS(LOSO) 17

A.10 PEDIATRIC LOW-GRADE GLIOMA (PLGG) AND DIFFUSE MIDLINE GLIOMA (DMG) . 17

A.11 ITMT AND SOCIAL DETERMINANTS OF HEALTH 18

SUPPLEMENTARY FIGURES 19

SUPPLEMENTARY TABLES 40

SUPPLEMENTARY REFERENCES 47

List of abbreviations:

LMM - Lean muscle mass

iTMT - Deep learning temporalis muscle thickness

TMT - Temporalis muscle thickness

DL – Deep learning

MRI - Magnetic resonance imaging

BMI - Body mass index

CDC - Centers for Disease Control and Prevention

WHO - World Health Organization

DXA - Dual-energy x-ray absorptiometry

CT - Computed tomography

CSA - Cross-sectional area

MAE - Mean absolute error

Dice/DSC - Dice similarity coefficient

IQR - Interquartile range

95%CI – 95% confidence interval

GAMLSS - Generalized Additive Models for Location Scale and Shape

DHEA - Dehydroepiandrosterone (DHEA)

pLGG - Pediatric Low-Grade Glioma

DMG – Diffuse midline glioma

Supplementary Methods

A.1 Protocol for Ground Truth Annotation

The temporalis muscle segmentation was defined as the 2D axial-plane volume of the temporalis muscle visible on T1-weighted (+/- contrast) MRI at the level of the superior edge of the orbital roof. Temporalis muscle segmentation was annotated using ITK-SNAP v4.0 [1]. All readers were blinded to the demographic and clinical characteristics of patients. To decrease the labor-intensive workload of TM annotation, we initially trained a model on a smaller set of annotations and then perform pseudo-labeling with the weakly trained segmentations. All pseudo-labeled segmentations were inspected and corrected where necessary and final models were retrained using the entire dataset.

1. Download ITK-SNAP [http://www.itksnap.org/pmwiki/pmwiki.php].
2. For each MRI scan, check registration and mark it in the file as Y-Yes/N-No. Do not annotate if the registration is bad (Figure S1-Figure S2).

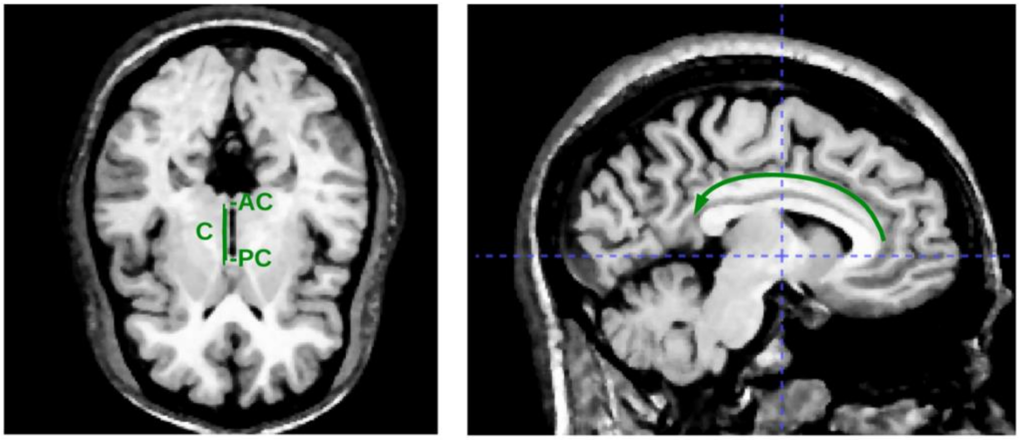

Figure S1 Example of "good" registration – AC-PC line visible

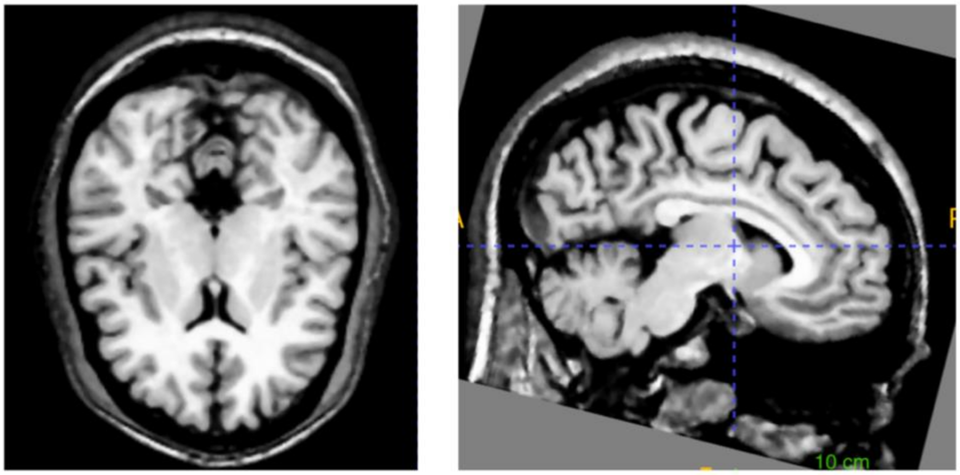

Figure S2 Example of "bad" registration, AC-PC line not visible

3. Select the correct slice – one above the top orbital roof.

4. Annotate the 2 TM muscles separately on one slice:

4.1. On the Main toolbar, click "polygon" (or press 3 on the keyboard)

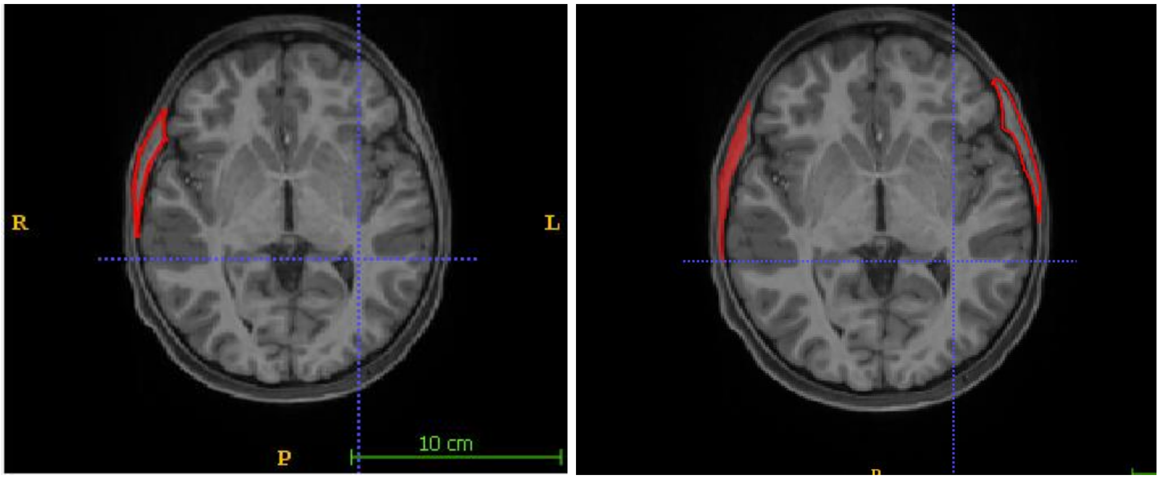

Figure S3 ITK-Slicer: Polygon segmentation mode

4.2. Start with placing the initial annotation by using mouse and left-click

4.3 Keep clicking around the TM until you reach the starting point (Figure S3)

4.4 Press enter and save segmentation by ctrl+S and give name "TM.nii.gz"

A.2 Head-circumference iTMT Adjustment

The measurement of maximal head circumference is a standard procedure in the examination of children's cranial growth and brain development [2]. We measured the correlation between iTMT and found that head circumference moderately correlates with temporalis muscle growth (Pearson r=0.42). We created an automated procedure for the head circumference measurement by performing a median filter on an N-dimensional array using Python SciPy package, applying a binary filter, and using the findContours function from the Python OpenCV package (Figure S7-Figure S8).

A.3 Feret Diameter

We quantified the TM using the Feret diameter Python implementation [3], a method for measuring projections of a 3D object into 2D space in microscopy. Another advantage of using the Feret diameter is that it is not affected by muscle orientation and is robust, unlike a human subjective definition based on the marker location, which can be a concern with other methods of measurement (Figure S9).

A.4 Model Sensitivity Analysis

The median model TM cross-sectional area error was lower in healthy children (53.3±36.9 mm^2) compared to those with brain tumors (89.5±79.9 mm^2, Table S1, Figure S*10*).

On average, one iTMT centile point change translated to 0.296 mm (for females mean=0.149mm [95%CI: 0.124-0.173]; for males mean=0.176mm [95%CI: 0.15-0.2], see Table S2 for age and sex averages and

Table *S3* for the impact of slice selection model error on the accuracy of CSA and temporalis muscle thickness (TMT)).

Given the MAE of 1.06 mm, we estimated that iTMT had precision within 6 centiles [95%CI:5.3-7] for males and 7.11 centiles for females [95%CI:6.1-8.5]. We found that both inter-expert and model-expert agreement improved with age, likely owing to larger and more robustly identifiable temporalis muscles in adolescents and young adults (Figure S11, Figure S12).

We compared two post-processing techniques that can be applied after UNet segmentation to improve accuracy. The first is Major Voting (MV), where each temporalis muscle is permuted 4 times and model predictions are combined via major voting. This means each pixel prediction is only deemed valid if it is present in 3 out of the 4 predictions. The second technique is Islands Filtering (IF). Here, the volumes of the predicted masks are computed and only the bigger mask volume is used for calculating the final thickness. The best result combining both methods achieved an average MAE TMT 1.05 mm (Figure S*35*, Table S14).

A.5 MRI Quality Assessment

To access MRI quality in the test cohort, we ran MRIQC [4] docker container and did the feature importance analysis by sorting the computed scan-wise correlations with iTMT MAE error. We analyzed the correlation between the two highest correlated features: qi_2 (is the proportion of voxels with intensity corrupted by artifacts normalized by the number of voxels in the background [5]) and rpve_gm (the residual partial volume effect of central spinal fluid (CSF), gray matter (GM) and white matter (WM)). We found out that higher rpve_gm (R=0.32) is associated with higher iTMT error, and lower qi_2(R=0.24) has a negative correlation with iTMT MAE error (Figure S13).

A.6 GAMLSS Model Diagnostics

Since we did not want to assume that the trajectories would automatically or by extension, be best captured by the identical underlying distributions, we decided to evaluate all possible distribution families. To choose between the Box-Cox Cole and

Green (BCCGo), Box-Cox Power Exponential (BCPEo), and Box-Cox t (BCTo) distributions, we use the generalized Akaike information criterion GAIC(k). GAIC(2) is the standard AIC, while GAIC (log n) is the Bayesian Information Criterion(BIC)[6]. According to the GAIC and BIC, the BCTo distribution is best for both models (see Table S5 for the males' model and Table S6 for females). Figure S16 presents the centile curves for the BCCGo, BCPEo, and BCTo distributions for the males' iTMT model and Figure S14 for females' iTMT model; Figure S17 for the males' iCSA model, and Figure S15 for the females' iCSA model. The reason for using high n for the criterion (i.e. k=log(12358)) is to achieve smooth centiles; a lower value of k (e.g. k = 4) would result in less smooth centiles but a better fit to the data(Table S5-Table S6).

Detrended transformed Owen's plots (DTOPs) is a diagnostic tool for checking whether the normalized quantile residuals come from a normal distribution(Figure S18). DTOPs have a slight advantage over the traditional Q-Q (quantile-quantile) plots of being a more flexible form of the distribution and thus provide a way to compare goodness-of-fit across different distributions (see Figure S19 for iTMT females model, Figure S20 for iCSA females model, Figure S21 for iTMT males model, Figure S22 for iCSA males model). Neither approach alone is definitive for assessing GAMLSS fits, and Stasinopoulus[6,7] recommends various approaches, including both Q-Q plots and DTOPs.

Residual distribution measures showed that residuals were normally distributed and highly correlated with model-predicted normality: iTMT Males model (mean=0.002, variance=1.0004, skewness=-0.019, kurtosis=3.117, Filliben probability plot correlation coefficient=0.99), iTMT Females model (mean=0.004, variance=1.0002, skewness=-0.025, kurtosis=3.22, Filliben probability plot correlation coefficient =0.99)

With the GAMLSS implementation outlined above, we optimized the choice of model parameters, including the outcome distribution and choice of fixed and random effects. In addition, GAMLSS provides automated parameter optimization to obtain the best fit given the included data, covariates, and random effects (Figure S23-Figure S28).

A.7 Primary Datasets

a) Model development datasets

For the model training and testing, we annotated a dataset with patients aged 3 to 35 from 7 different imaging centers, stratified by age/sex, and performed data augmentation for each DL stage separately (327 MRI T1w unique patients, UNet training: 1120 images, DenseNet training: 45695 images). 3 datasets were used for training and validation: Pediatric MRI demographic NIMH [8], ABCD [9], and IXI[10]; and 3 datasets were used for testing as a healthy cohort: HBN [11], Pixar [12], and Baby Connectome project [13]; and 1 data set of patients with brain tumors, CBTN[14] (see for the MRI scanning protocols and details on the demographics data). The two-stage temporalis muscle auto-segmentation pipeline was evaluated on a test set of 46 healthy subject MRIs (age IQR 6-19y, mean 13.3y, 94 TMs) and 25 MRIs of patients with brain tumors (age IQR 7.3-14.8y, mean 10.8y, 64 TMs) curated from four data sources, all external to the algorithm development sources(Figure S29).

b) Acceptability testing dataset

For the acceptability testing pipeline, we curate 2950 images from the aggregated dataset (23,852 MRIs, 13 datasets, Table S7-Table S8) stratified by age/sex. Two reviewers checked and assigned a rating from 1 to 4 using a Likert-type score (1: Acceptable, no changes; 2: Acceptable, minor changes; 3: Unacceptable, major changes; 4: Unacceptable, not visible/wrong location). We binned the Likert-type score into two groups (1+2: Accepted; 3+4: Not accepted) and computed inter-rater agreement using Gwet AC1[15] (0.94) due to the significant class imbalance. We asked a third reader(tiebreaker) to review 159 challenging cases where two annotators disagreed and computed inter-rater agreement between Rater 1 and the tiebreaker was 0.96; between Rater 2 and the tiebreaker 0.98.

c) iTMT Growth Chart Datasets

For the centile charts, we curated 23,852 MRIs from 13 datasets (ABCD[9], ABIDE [16], AOMIC[17], Baby Connectome[13], Calgary[18], ICBM[19], IXI[10], NIMH[8], PING[20], Pixar[12],

SALD[21], NYU2(CoRR) [22], Healthy Adults[23]; 90% United States; 52% male; 26% White, 6% Hispanic, 5% Black, 2% Asian, 4% Mixed, 1% Other, 56% Unknown, Table S7). We further split aggregated data by sex and remove zeros TMT predictions (less than 1%, upon further investigation, these are corrupted MRI scans which suggests a need for an automatic MRI quality assessment), resulting in 12,358/11,336 male/female observations. For the ethnicity- and sex-specific charts, we used ABCD[9] dataset (Figure S*30*).

To measure dataset variability, we conduct a leave-one-out analysis and mean analysis (Figure S31-Figure S*33*). The greater variation in standardized mean TMT scores observed in some data sources and age groups can be attributed to smaller subset sample sizes. With fewer subjects in a given age range for certain sources, the mean TMT calculation may have relied on just one or two individuals' scores.

ABCD

Data used in the preparation of this article were obtained from the Adolescent Brain Cognitive Development SM (ABCD) Study (https://abcdstudy.org), held in the NIMH Data Archive (NDA). This is a multisite, longitudinal study designed to recruit more than 10,000 children age 9-10 and follow them over 10 years into early adulthood. The ABCD Study® is supported by the National Institutes of Health and additional federal partners under award numbers U01DA041048, U01DA050989, U01DA051016, U01DA041022, U01DA051018, U01DA051037, U01DA050987, U01DA041174, U01DA041106, U01DA041117, U01DA041028, U01DA041134, U01DA050988, U01DA051039, U01DA041156, U01DA041025, U01DA041120, U01DA051038, U01DA041148, U01DA041093, U01DA041089, U24DA041123, U24DA041147. A full list of supporters is available at abcdstudy.org [https://abcdstudy.org/federal-partners.html]. A listing of participating sites and a complete listing of the study investigators can be found at abcdstudy.org/consortium_members [https://abcdstudy.org/consortium_members/]. ABCD consortium investigators designed and implemented the study and/or provided data but did not necessarily participate in the analysis or writing of this report. This manuscript reflects the authors' views and may not reflect the opinions or views of the NIH or ABCD consortium investigators.

The ABCD data repository grows and changes over time. The ABCD data used in this report came from the fast-track data release. The raw data are available at NDA [https://nda.nih.gov/edit_collection.html?id=2573]).
Additional support for this work was made possible from supplements to U24DA041123 and U24DA041147, the National Science Foundation (NSF 2028680), and Children and Screens: Institute of Digital Media and Child Development Inc. [9]

ABIDE

ABIDE II involves 19 sites, ten charter institutions and seven new members, overall donating 1114 datasets from 521 individuals with ASD and 593 controls (age range: 5-64 years). These data were openly released to the scientific community on June 2016. In accordance with HIPAA guidelines and 1000 Functional Connectomes Project / INDI protocols, all datasets are anonymous, with no protected health information included. Consistent with its popularity in the imaging community and prior usage in FCP/INDI efforts, the NIFTI format was selected to store the ABIDE II MRI datasets [http://fcon_1000.projects.nitrc.org/indi/abide/abide_II.html]. With the exception of a single collection (IP1, 1.5 Tesla), all MRI data were acquired using 3 Tesla scanners[16].

Aomic

The Amsterdam Open MRI Collection (AOMIC [https://openneuro.org/datasets/ds003097/versions/1.2.1]) is a collection of three datasets with multimodal (3T) MRI data, including structural (T1-weighted), diffusion-weighted, and (resting-state and task-based) functional BOLD MRI data, as well as detailed demographics and psychometric variables from a large set of healthy participants (N = 928, N = 226, and N = 216). Data from all three datasets were acquired on the same Philips 3T scanner (Philips, Best, the Netherlands) but underwent several upgrades in between the three studies [17].

Baby Connectome

The Baby Connectome Project (BCP [https://nda.nih.gov/edit_collection.html?id=2848]) is a four-year study of children from birth through five years of age, intended to provide

a better understanding of how the brain develops from infancy through early childhood and the factors that contribute to healthy brain development. This project is a research initiative of the Neuroscience Blueprint – a cooperative effort among the 15 NIH Institutes, Centers, and Offices that support neuroscience research. The BCP is supported by Wyeth Nutrition through a donation to the FNIH. Images are acquired on 3T Siemens Prisma MRI scanners using a Siemens 32-channel head coil at the Center for Magnetic Resonance Research (CMRR) at the University of Minnesota and the Biomedical Research Imaging Center (BRIC) at the University of North Carolina at Chapel Hill [13].

Calgary

The Preschool MRI study in The Developmental Neuroimaging Lab at the University of Calgary uses different magnetic resonance imaging (MRI) techniques to study brain structure and function in early childhood (OSF [https://osf.io/axz5r/files/osfstorage]). All imaging for this dataset was conducted using the same General Electric 3T MR750w system and 32-channel head coil (GE, Waukesha, WI) at the Alberta Children's Hospital in Calgary, Canada. Children were scanned either while awake and watching a movie, or while sleeping without sedation. The University of Cal- gary Conjoint Health Research Ethics Board (CHREB) approved this study (REB13-0020). T1-weighted images were acquired using an FSPGR BRAVO sequence with TR = 8.23 ms, TE = 3.76 ms, TI = 540 ms, flip angle=12 degrees, voxel size = 0.9x0.9x0.9 mm3, 210 slices, matrix size=512x512, field of view=23.0 cm. ASL images were acquired with the vendor supplied pseudo continuous 3D ASL sequence with TR = 4.56 s, TE = 10.7 ms, in-plane reso- lution of 3.5x3.5 mm2, post label delay of 1.5 s, and thirty 4.0 mm thick slices. The sequence scan time was 4.4 minutes [18]

ICBM

Data used in the preparation of this work were obtained from the International Consortium for Brain Mapping (ICBM) database (ICBM [www.loni.usc.edu/ICBM]). The ICBM project (Principal Investigator John Mazziotta, M.D., University of California, Los Angeles) is supported by the National Institute of Biomedical Imaging and

BioEngineering. ICBM is the result of efforts of co-investigators from UCLA, Montreal Neurologic Institute, University of Texas at San Antonio, and the Institute of Medicine, Juelich/Heinrich Heine University - Germany. Data collection and sharing for this project was provided by the International Consortium for Brain Mapping (ICBM; Principal Investigator: John Mazziotta, MD, PhD). ICBM funding was provided by the National Institute of Biomedical Imaging and BioEngineering. ICBM data are disseminated by the Laboratory of Neuro Imaging at the University of Southern California [19].

IXI

The data [https://brain-development.org/ixi-dataset/] has been collected at three different hospitals in London:Hammersmith Hospital using a Philips 3T system (details of scanner parameters [http://brain-development.org/scanner-philips-medical-systems-intera-3t/]), Guy's Hospital using a Philips 1.5T system (details of scanner parameters [http://brain-development.org/scanner-philips-medical-systems-gyroscan-intera-1-5t/]), Institute of Psychiatry using a GE 1.5T system (details of the scan parameters not available at the moment). The Thames Valley MREC granted ethical approval. The T1 and T2 images were acquired prior to diffusion-weighted imaging using 3D MRPRAGE and dual-echo weighted imaging [10].

NIMH

The data used in this work was collected from the 5.1 release [https://nda.nih.gov/edit_collection.html?id=1151] . MRI scans were acquired using either General Electric or Siemens 1.5 Tesla scanners involving six sites or Pediatric Study Centers (PSC) in the United States. The Institutional Review Board at the University of Wisconsin-Madison also approved the analysis of the data of this human subject. Sequence type: 3D FLASH/SPGR; GE sequence: pulse sequence=SPGR, mode=3D; TR: 22 ms; TE: 10-11 ms; excitation pulse angle: 30 degrees; orientation: sagittal; FoV: 250mmISx250mmAP; matrix: 256 x 256 (x 124 - 180 slices); slices: 160-180 slices of 1-1.5 mm thickness (cover entire head). Note that on GE systems with a

124-slice limitation, slice thickness should be adjusted to cover the entire head with 124 slices: signal averages: 1; scan time: 11.6 – 16.8 min [8].

PING

The PING Data Resource [https://nda.nih.gov/edit_collection.html?id=2607] is the product of a multi-site project involving developmental researchers across the United States, including UC San Diego, the University of Hawaii UC Los Angeles Children's Hospital of Los Angeles of the University of Southern California UC Davis Kennedy Krieger Institute of Johns Hopkins University Sackler Institute of Cornell University University of Massachusetts Massachusetts General Hospital at Harvard University and Yale University. The Data Resource includes neurodevelopmental histories, information about developing mental and emotional functions, multimodal brain imaging data, and genotypes for well over 1000 children and adolescents between the ages of 3 and 20. The PING imaging protocol takes advantage of key technologies developed for the consortium and builds on earlier methods development performed as part of the Biomedical Informatics Research Network (BIRN [24] and the Alzheimer's Disease Neuroimaging Initiative (ADNI [25]). Specifically, a standard PING scan session included: 1) a 3D T1-weighted inversion prepared RF-spoiled gradient echo scan using prospective motion correction (PROMO), for cortical and subcortical segmentation; 2) a 3D T2-weighted variable flip angle fast spin echo scan, also using PROMO, for detection and quantification of white matter lesions and segmentation of CSF; 3) a high angular resolution diffusion imaging (HARDI) scan, with integrated B0 distortion correction (DISCO), for segmentation of white matter tracts and measurement of diffusion parameters; and 4) a resting state blood oxygenation level-dependent (BOLD) fMRI scan, with integrated distortion correction. Pulse sequence parameters used across (3 T) scanner manufacturers (GE, Siemens, and Phillips) and models were optimized for equivalence in contrast properties and consistency in image-derived quantitative measures [20].

Pixar

One hundred twenty-two 3.5–12-year-old children (M(s.d.) = 6.7(2.3); 64 females) participated in the study (Openfmri [https://openfmri.org/dataset/ds000228/]). Child and adult participants were recruited from the local community. All adult participants gave written consent; parent/guardian consent and child assent was received for all child participants. Recruitment and experiment protocols were approved by the Committee on the Use of Humans as Experimental Subjects (COUHES) at the Massachusetts Institute of Technology. Whole-brain structural and functional MRI data were acquired on a 3-Tesla Siemens Tim Trio scanner located at the Athinoula A. Martinos Imaging Center at MIT. Children under age 5 years used one of two custom 32-channel phased-array head coils made for younger (n = 3, M(s.d.) = 3.91(.42) years) or older (n = 28, M(s.d.) = 4.07(.42) years) children; all other participants used the standard Siemens 32-channel head coil. T1-weighted structural images were collected in 176 interleaved sagittal slices with 1 mm isotropic voxels (GRAPPA parallel imaging, acceleration factor of 3; adult coil: FOV: 256 mm; kid coils: FOV: 192 mm). Functional data were collected with a gradient-echo EPI sequence sensitive to Blood Oxygen Level Dependent (BOLD) contrast in 32 interleaved near-axial slices aligned with the anterior/posterior commissure and covering the whole brain (EPI factor: 64; TR: 2 s, TE: 30 ms, flip angle: 90°). This data was obtained from the OpenfMRI database, accession number is ds000228. Dataset version 1.0.2 [12]

SALD

The data was generated in the Southwest University Adult Lifespan Dataset (SALD [http://fcon_1000.projects.nitrc.org/indi/retro/sald.html]), which comprises a large cross-sectional sample (n = 494; age range = 19-80) undergoing a multi-modal (sMRI, rs-fMRI, and behavioral). All data were collected at the Southwest University Center for Brain Imaging using a 3.0-T Siemens Trio MRI scanner (Siemens Medical, Erlangen, Germany). A magnetization-prepared rapid gradient echo (MPRAGE) sequence was used to acquire high-resolution T1-weighted anatomical images (repetition time=1,900ms, echo time=2.52ms, inversion time=900ms, flip angle=90 degrees, resolution matrix=256×256, slices=176, thickness =1.0mm, and voxel size=111mm3) [21].

NYU2(CoRR)

The Consortium for Reliability and Reproducibility (CoRR [http://fcon_1000.projects.nitrc.org/fcpClassic/FcpTable.html]) has aggregated 1,629 typical individuals' resting state fMRI (rfMRI) data (5,093 rfMRI scans) from 18 international sites and is openly sharing them via the International Data-sharing Neuroimaging Initiative (INDI). In this study, we used a subset from CoRR study "NYU 2" created by New York University (Di Martino, Kelly)[26].

Healthy adults

The dataset was collected and shared under the NIMH Healthy Research Volunteer (RV) Study (Recruitment and Characterization of Healthy Research Volunteer for NIMH Intramural Studies NCT033046, [https://openneuro.org/datasets/ds004215/versions/1.0.1]). Data collection is ongoing, while data from 1,090 participants (155 with MRI) is shared. The MR protocol used was initially based on the ADNI-3 basic protocol, but was later modified to include portions of the ABCD protocol. Because there may be small changes in parameters from the standard ABCD/ADNI3 sequences, detailed sequence descriptions are shared in the BIDS source data directory. [23].

28andMe

In Study 1 (sessions 1-30, 2018), the female participant was naturally cycling; in Study 2 (sessions 31-60, 2019), the participant was placed on an oral hormonal contraceptive regimen (OpenNeuro [https://openneuro.org/datasets/ds002674/versions/1.0.5/download]) . The participant underwent a daily magnetic resonance imaging scan on a Siemens 3T Prisma scanner equipped with a 64-channel phased-array head coil. First, high-resolution anatomical scans were acquired using a T1-weighted magnetization prepared rapid gradient echo (MPRAGE) sequence (TR = 2500 ms, TE = 2.31 ms, TI = 934 ms, flip angle = 7º, 0.8 mm thickness) followed by a gradient echo fieldmap (TR = 758 ms; TE1 = 4.92 ms; TE2 = 7.38 ms; flip angle = 60º). Next, the participant completed a 10-minute resting-state fMRI scan using a T2*- weighted multi-band echo-planar imaging (EPI) sequence

sensitive to the blood oxygenation level-dependent (BOLD) contrast (72 oblique slices, TR = 720 ms, TE = 37 ms, voxel size = 2 mm3, flip angle = 56º, multiband factor = 8). High-resolution anatomical scans were acquired using a T1-weighted magnetization prepared rapid gradient echo (MPRAGE) sequence (TR = 2500 ms, TE = 2.31 ms, TI = 934 ms, flip angle = 7º, 0.8 mm thickness) followed by a gradient echo fieldmap (TR = 758 ms; TE1 = 4.92 ms; TE2 = 7.38 ms; flip angle = 60º). A T2- weighted turbo spin echo (TSE) scan was also acquired with an oblique coronal orientation positioned orthogonally to the main axis of the hippocampus (TR/TE= 8100/50 ms, flip angle = 122°, 0.4 × 0.4 mm2 in plane resolution, 2 mm slice thickness, 31 interleaved slices with no gap, total acquisition time = 4:21 min) [27].

BCH Dataset

This dataset includes MRI scans curated from Boston Children's' Hospital, Boston, MA. All data was de-identified for the purposes of the study, and thus classified as non-human subject research. As such, such waiver of consent was granted by the IRB. This dataset contains private hospital data that is controlled due to privacy concerns. Access to the derived dataset will be considered upon reasonable request to the corresponding author. Multimodal MRI scans were acquired using either 1.5 Tesla or 3.0 Tesla scanners from the following manufacturers and models: Siemens (Verio, Skyra, TrioTim, Symphony, Avanto, Sonata, Spree), GE Medical System (Excite, Genesis,_Signa, Optima MR450w), Philips Medical Systems (Intera, Ingenia, Achieva, Panorama HFO), Toshiba Titan and Hitachi Altaire. A magnetization-prepared rapid gradient echo (MPRAGE) sequence or a turbo spin echo sequence was used to acquire high-resolution T1-weighted anatomical images (repetition time = 4 – 3400 ms, echo time = 1.35 – 99 ms, flip angle=2-180 degrees, slice thickness=0.74-6.0 mm, and voxel size=0.35 – 1.33 mm3).

CBTN Dataset

This dataset includes MRI scans curated from the Children Brain Tumor Network [https://cbtn.org/]. Multimodality MRI scans were acquired using either 1.5 Tesla or 3.0 Tesla scanners with either a 20-channel or 30-channel phased-array head coil from the following manufacturers and models: Siemens Prisma, Siemens Skyra, Siemens

TrioTim, Siemens Avanto. A magnetization-prepared rapid gradient echo (MPRAGE) sequence or a turbo spin echo sequence was used to acquire high-resolution T1-weighted anatomical images (repetition time = 8 – 2560 ms, echo time = 2 – 86 ms, flip angle=8-150 degrees, slice thickness=1-5 mm, and voxel size=0.35 - 1 mm3).

A.8 Physical Biomarkers Definitions

BMI normal reference tables were downloaded from the CDC[28]. For daily nutrition according to dietary recommendations, healthy child kcal/d intake for range 9-13 years for female 1600 kcal/d, male 1800 kcal/d. Therefore, as suggested in [29], we define a normal healthy child kcal/d intake for female in range [1400-2200]; for male [1600-2600]. For daily activity level, authors in [30] suggest that males to average 12,000 to 16,000 steps/day and females to average 10,000 to 13,000 steps/day. HDL Cholesterol in healthy kids younger <18 should be more than 45 mg/dl; non-HDL <120mg/dL [31] (Table S*9*-Table S12)

A.9 Leave-one-study Out Analysis(LOSO)

Although the current sample is a large structural neuroimaging dataset, much of this data is derived from the ABCD study. To test whether our model's reliability was skewed, we performed a leave-one-study-out (LOSO) analysis. Specifically, we iteratively re-estimated all model parameters and extracted the fitted trajectories leaving out the ABCD cohort (Figure S*32*) and iteratively leaving out each of the studies (Figure S*33*). We compared the resulting fits for consistency by comparing 95% confidence intervals. We observed minor differences between median LOSO GAMLSS fitted curves, with a better-fitted curve in the LOSO ABCD analysis.

A.10 Pediatric Low-Grade Glioma (pLGG) and Diffuse Midline Glioma (DMG)

We applied iTMT to the internal dataset of patients with pLGG collected at Boston Children's Hospital (n=144, median follow-up: 8.8 years, IQR=6.3-15.2 years, 56% Female). To ensure the TM segmentation and iTMT were accurate, quality assurance of segmentations was performed (~1.4% inaccurate predicted mask). We further refined

TMT segmentations by applying the following rule to mitigate the effect of ipsilateral craniotomy that is commonly present in pLGG: if the difference between left and right TMT is bigger than 50%, use the bigger one (9 cases in total met criteria, 6.25%). We plan to improve model performance by training on more challenging and variable cases, e.g., post-surgical MRI scans and those with ipsilaterally removed TM. Once iTMT has been applied, age- and sex-adjusted iTMT percentiles were computed for all patients and scan time points.

We curated a dataset from patients with DMG treated at Dana-Farber/Harvard Cancer Center between 2008-2022 (N=85, Age IQR=7.8-25y, mean=16.3y; Female 54%; 56% deceased). We similarly performed manual verification of segmentations (27% discarded: 28% due to the poor MRI resolution, 25% corrupted image, 16% wrong slice selected, 13% image artifacts, 9% inaccurate predicted mask, 6% missing/invisible TM on one size, 3% motion artifacts) applied iTMT to this dataset, and further used normalized centiles to determine patients' z-scores, which demonstrated that approximately a third of patients with pHGG had low iTMT scores (41 patients were in < 25 centiles, 31 in 25-75%, and 11 were over > 75%). For a small subset of patients with a BMI index (N=27) present, the Spearman correlation coefficient between BMI and iTMT was 0.48(p=0.01).

A.11 iTMT and social determinants of health

To measure the association of iTMT with social and demographic factors in children, we fitted univariable and multivariable linear regressions for iTMT percentile as a target variable, including total household income, highest parent education, insurance, country of birth for child and family(inside or outside USA), food insecurity and race/ethnicity (total of 1227 records, Multiple R-squared: 0.14, F=15.13 (p=3.9e-32). We conducted this analysis using the ABCD dataset, which captures rich metadata, and thus analyses were limited to the 8-13-year-old United States group, which may limit generalizability to other countries and age groups. We checked for collinearity with variance inflation factor values, all of which were <2 suggesting limited collinearity. We found that statistically significant variables were if the family was born in the USA, ethnicity, and parent education (Table S13, Figure S*34*). This observation could be related to the disparities found across different groups [32], and socioeconomic factors. Of

note, lower parental education level was associated with increased iTMT. This could be, in part, reflective of increased BMI observed in these cohorts or other social factors influencing nutrition and/or exercise habits [33,34], though these hypotheses require further investigation.

We generated ethnicity-specific GAMLSS curves for female and male children aged 8-13 only due to the limited demographic data availability in other datasets (Figure S*30*).

Supplementary Figures

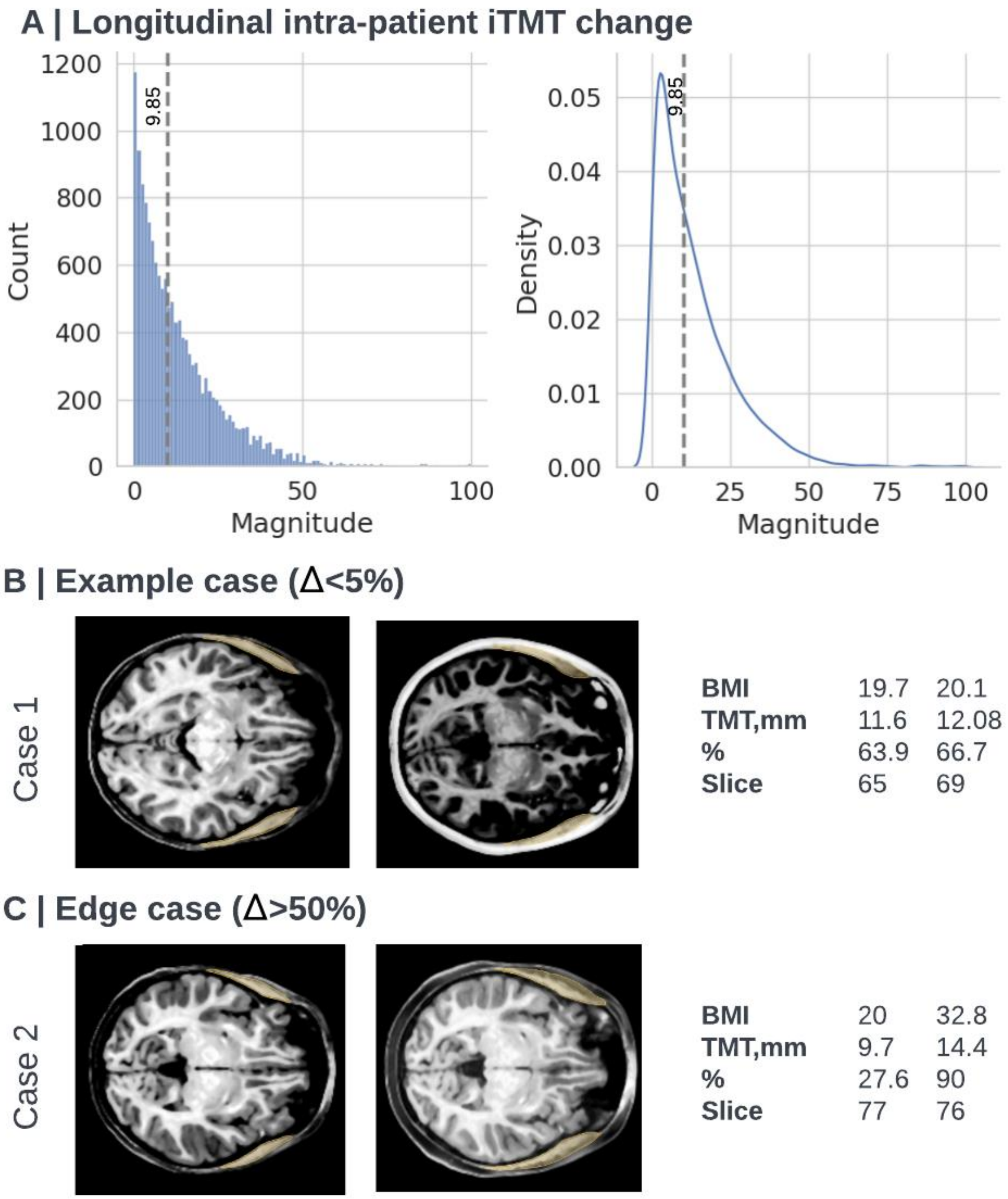

Figure S4 Longitudinal intra-patient study on the ABCD[9] cohort. A) We aggregated a dataset of 14642 patient visits with 2 MRI scans taken within two years and calculated their iTMT magnitude of change. The mean magnitude was 9.85 points. Less than <2% of patients experienced significant iTMT centile change (∆>50%), and 15.8% experienced minor iTMT change (∆>25%). B) Example subject with low iTMT change between two visits. C) Example subject with high iTMT and BMI change in between visits. TMT = temporalis muscle thickness, BMI = body mass index.

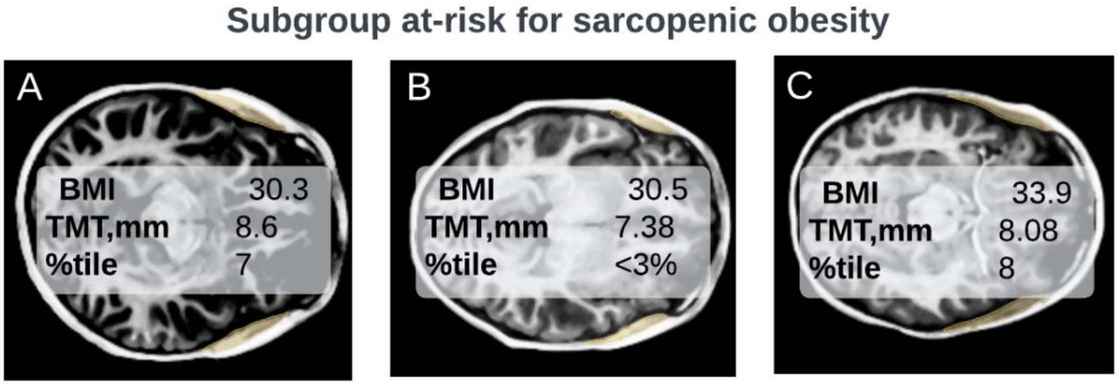

Figure S5. Three representative examples (A,B,C) from a low-iTMT, high-BMI subgroup, who may be at risk for sarcopenic obesity (Dataset: ABCD [9]).

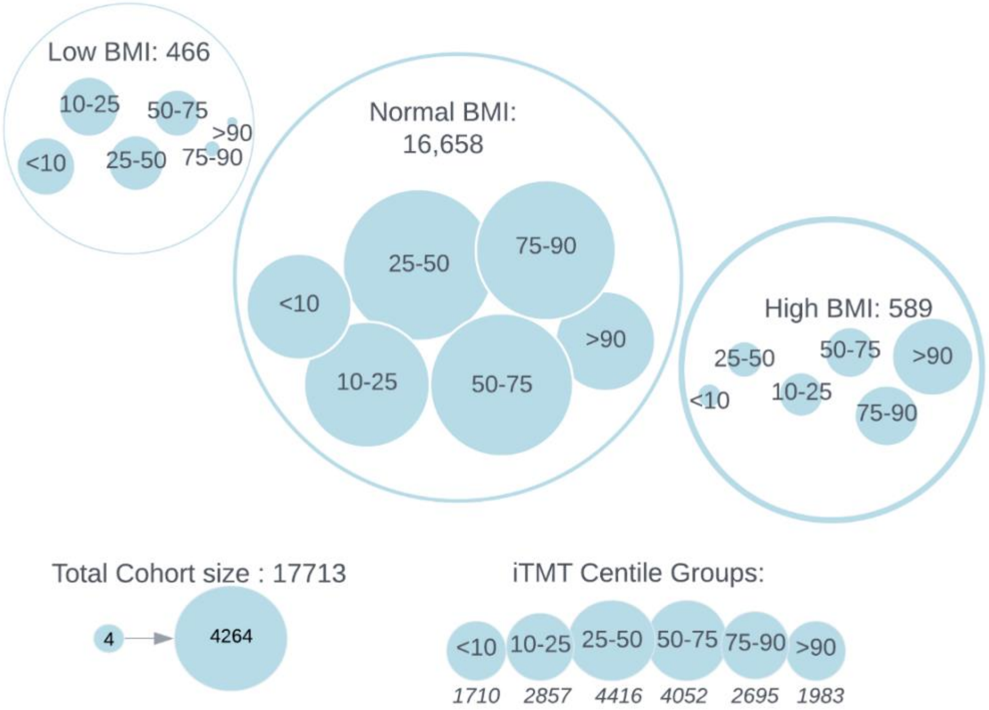

Figure S6 Subgroup cohort size analysis for iTMT grouped by BMI. The total number of MRI T1-weighted scans was 17,713; those with low BMI=466, normal BMI=16,658, and high BMI=589. BMI normal reference tables were downloaded from the CDC[28]. iTMT=automated temporalis muscle thickness, BMI=body mass index (Dataset: ABCD [9]).

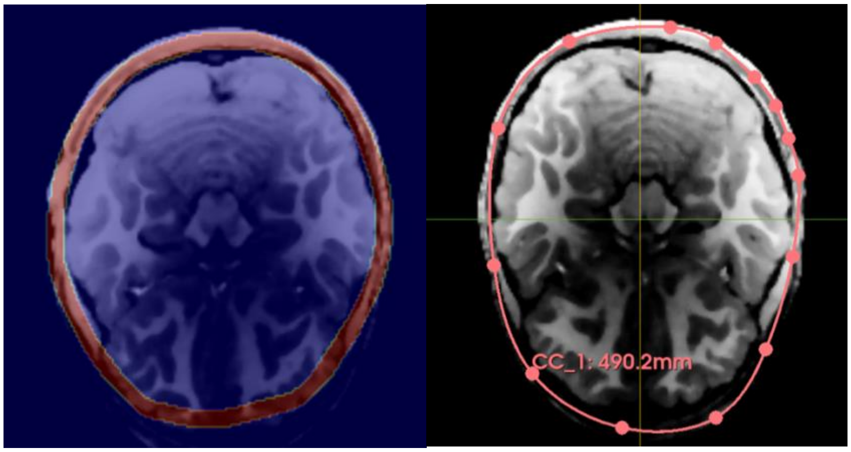

Figure S7. Comparison of the developed method using Python OpenCV(left) for head circumference measurement(480.0mm) compared to manual process (right, 490.2mm) in Slicer 3D[35].

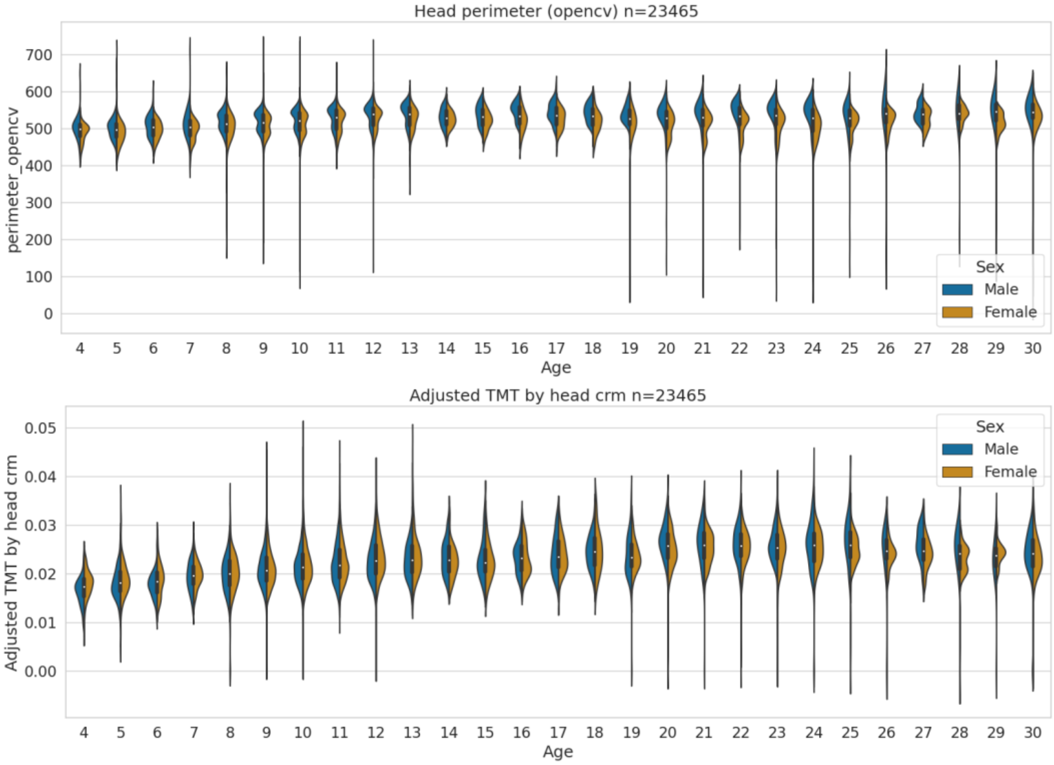

Figure S8. Top: violin plots head circumference for children and young adults aged 3-30 years (N=23465 MRI T1w scans). Violin plots show the kernel density estimation of the distribution, with a boxplot overlay, with the median marked by a white dot, the interquartile range marked by the thick black bar, and the range by the thin black line. Bottom: violin plots iTMT adjusted by head circumference. TMT = temporalis muscle thickness.

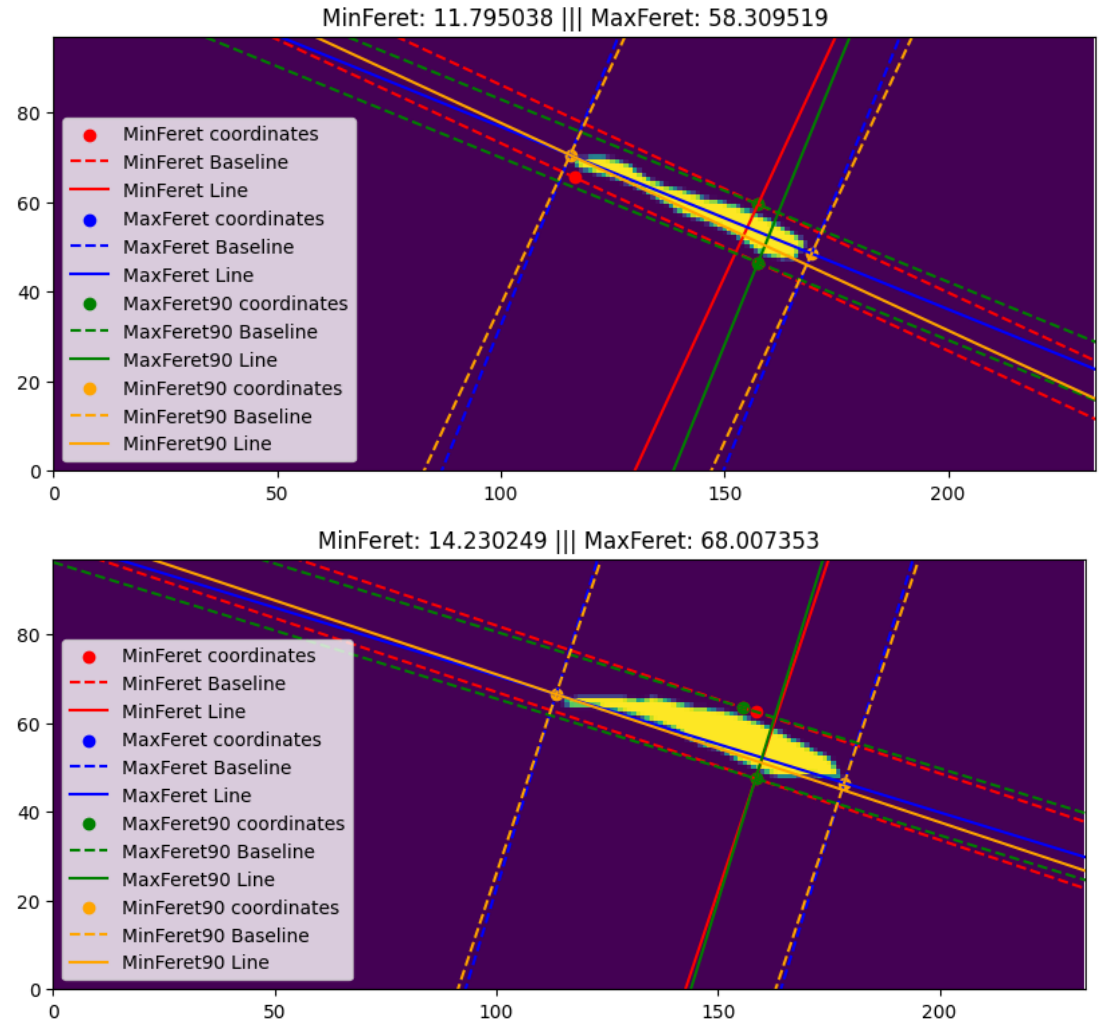

Figure S9 Examples of Feret diameter calculation. For the iTMT, we used minFeret calculation.

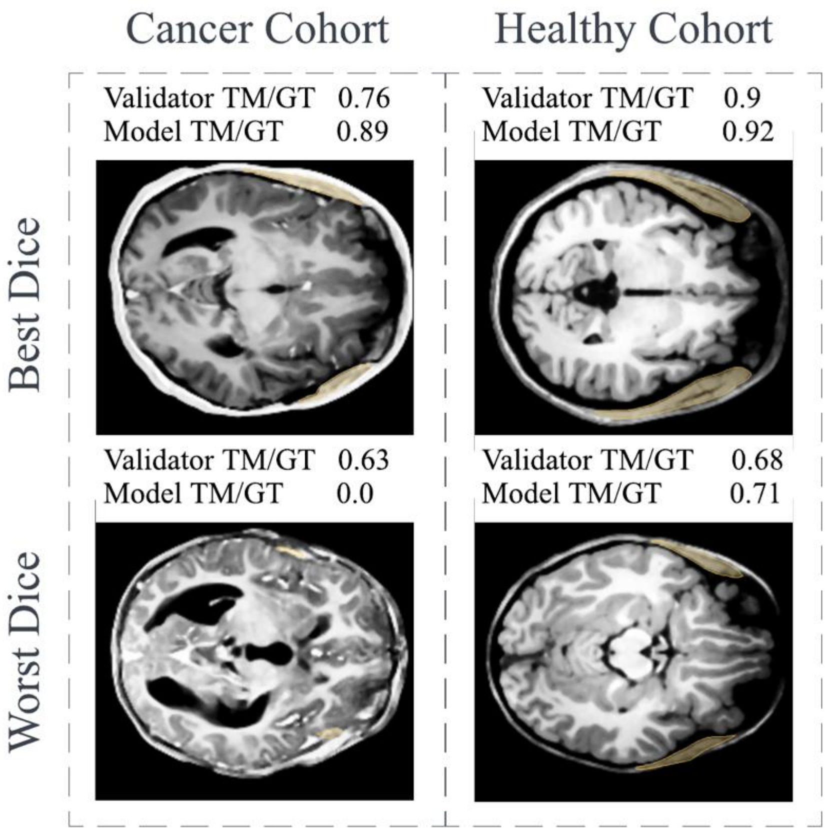

Figure S10 Example of model segmentations: best(top)/worst(bottom) Dice for the cohort with brain tumors(left) and healthy(right).

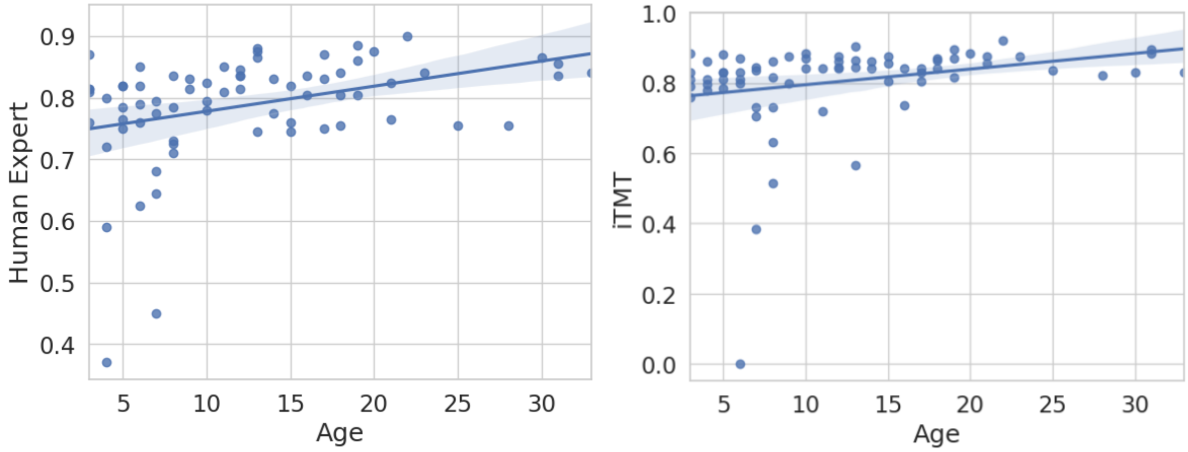

Figure S11. The distribution of disagreements (Dice) between Human Experts (left, Pearson's' correlation 0.35, p=0.002) and iTMT(right, Pearson's' correlation 0.26, p=0.02) by subject age on healthy and brain cancers cohorts(n=82). We found that both inter-expert and model-expert agreement improved with age, likely owing to larger and more robustly identifiable temporalis muscles in adolescents and young adults.

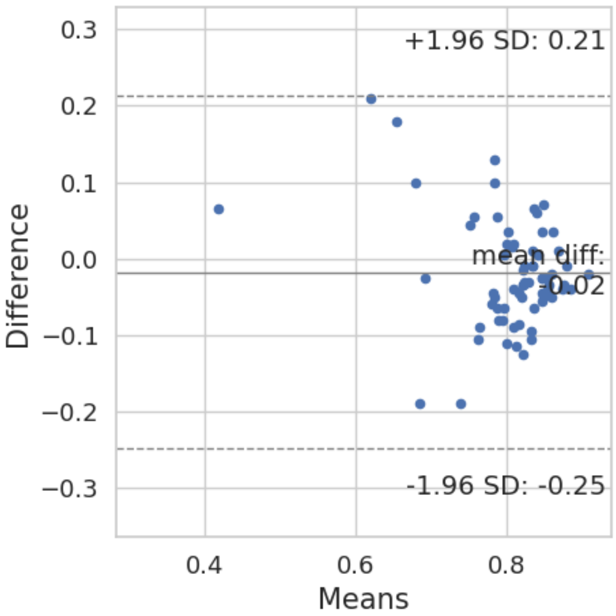

Figure S12 Bland-Altman plot shows agreement between iTMT and the human expert.

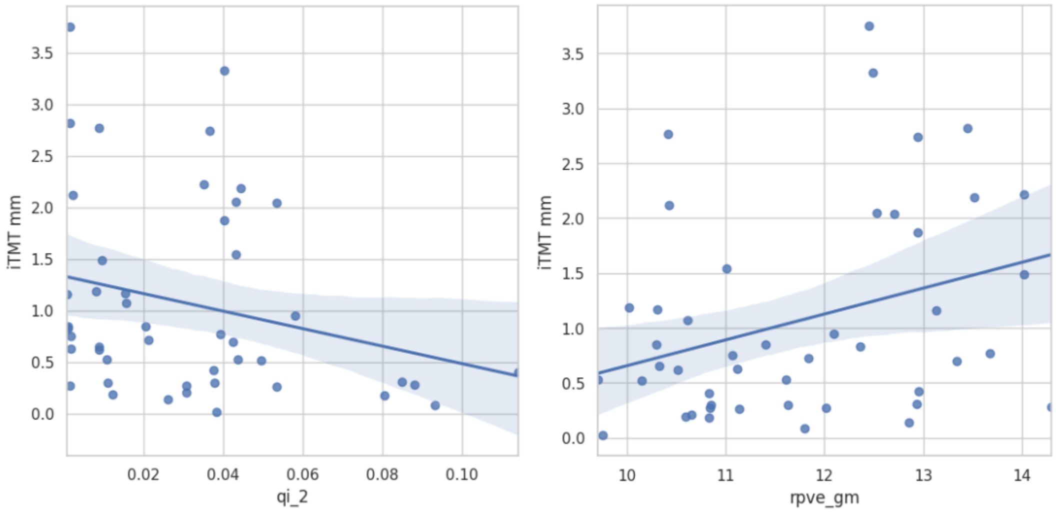

Figure S13 MR image quality metrics (MRIQC) extracted image quality characteristics and their correlation with the iTMT error (n=82). Higher rpve_gm (R=0.32) is associated with higher iTMT error, and lower qi_2(R=0.24) had a negative correlation with iTMT MAE error. iTMT = reproducible temporalis muscle thickness measurement, MAE = mean absolute error.

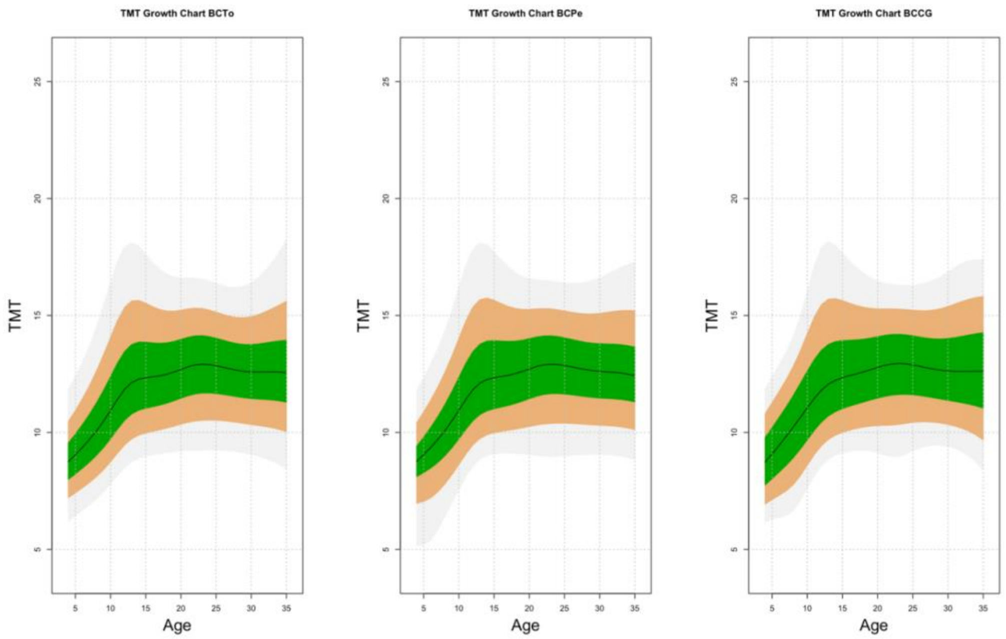

Figure S14 iTMT: Models females: comparison between Box-Cox Cole and Green (BCCGo), Box-Cox Power Exponential (BCPEo), and Box-Cox t (BCTo) . We choose BCTo distribution according to GAIC and BIC criterion. BIC= Bayesian Information Criterion; GAIC= generalized Akaike information criterion, iTMT = reproducible temporalis muscle thickness measurement.

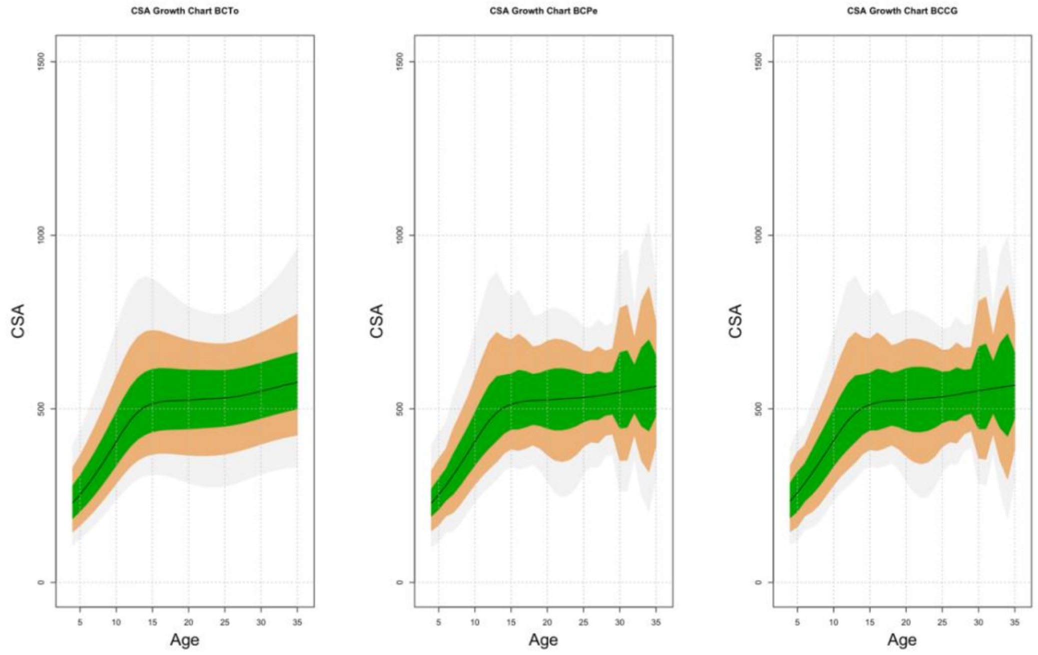

Figure S15 iCSA: Models females: comparison between Box-Cox Cole and Green (BCCGo), Box-Cox Power Exponential (BCPEo), and Box-Cox t (BCTo) . BIC= Bayesian Information Criterion; GAIC= generalized Akaike information criterion.

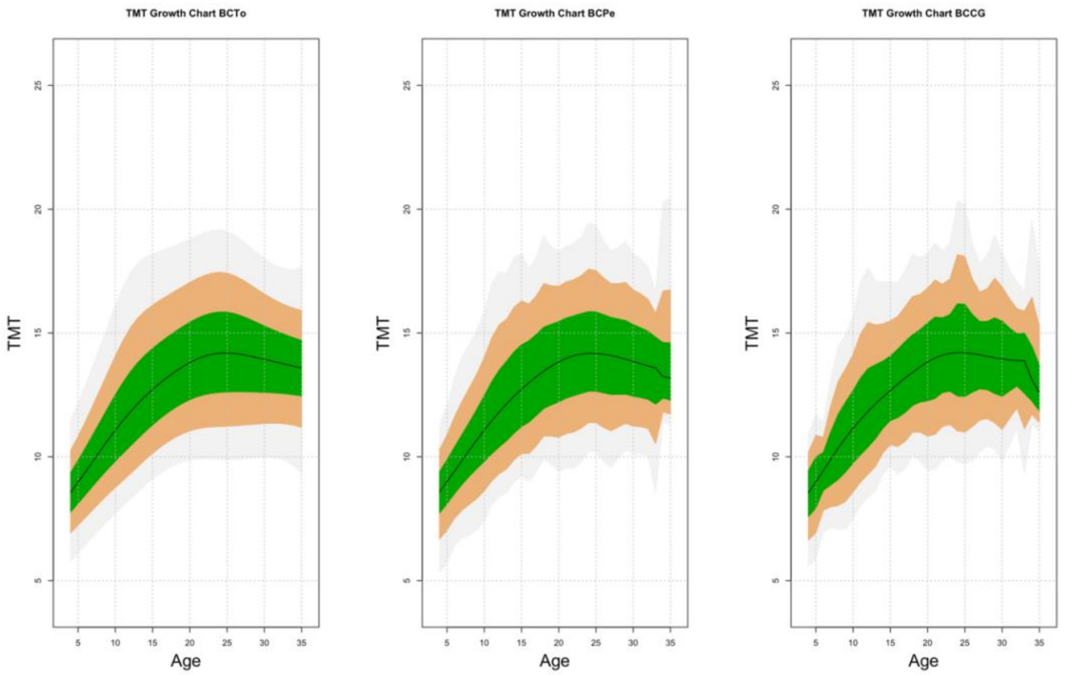

Figure S16 iTMT: Models males: comparison between Box-Cox Cole and Green (BCCGo), Box-Cox Power Exponential (BCPEo), and Box-Cox t (BCTo) . We choose BCTo distribution according to GAIC and BIC criterion. BIC= Bayesian Information Criterion; GAIC= generalized Akaike information criterion, iTMT = reproducible temporalis muscle thickness measurement.

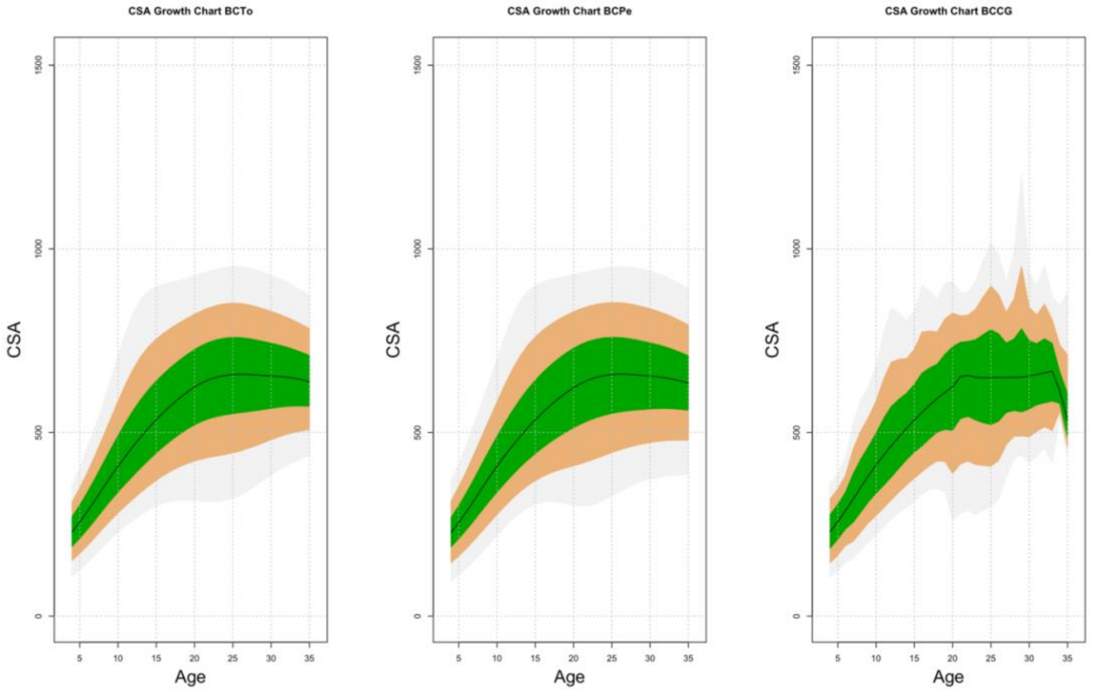

Figure S17 iCSA: Models males: comparison between Box-Cox Cole and Green (BCCGo), Box-Cox Power Exponential (BCPEo), and Box-Cox t (BCTo) . BIC= Bayesian Information Criterion; GAIC= generalized Akaike information criterion.

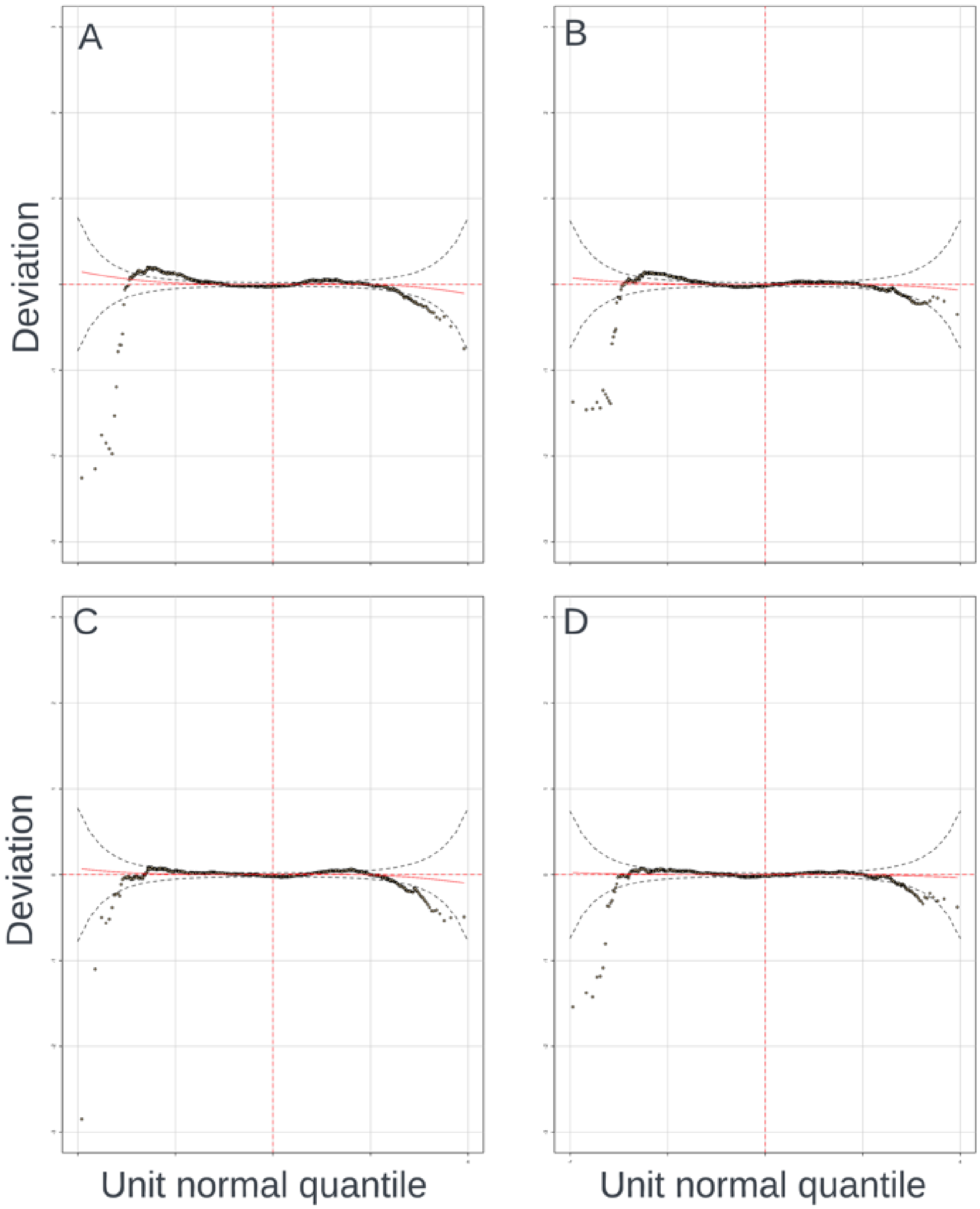

Figure S18 Detrended transformed Owen's plots of model residuals for males (A, C) and females (B, D) iTMT model (A, B) and iCSA (C, D). iTMT = reproducible temporalis muscle thickness measurement, iCSA = reproducible cross-sectional area measurement.

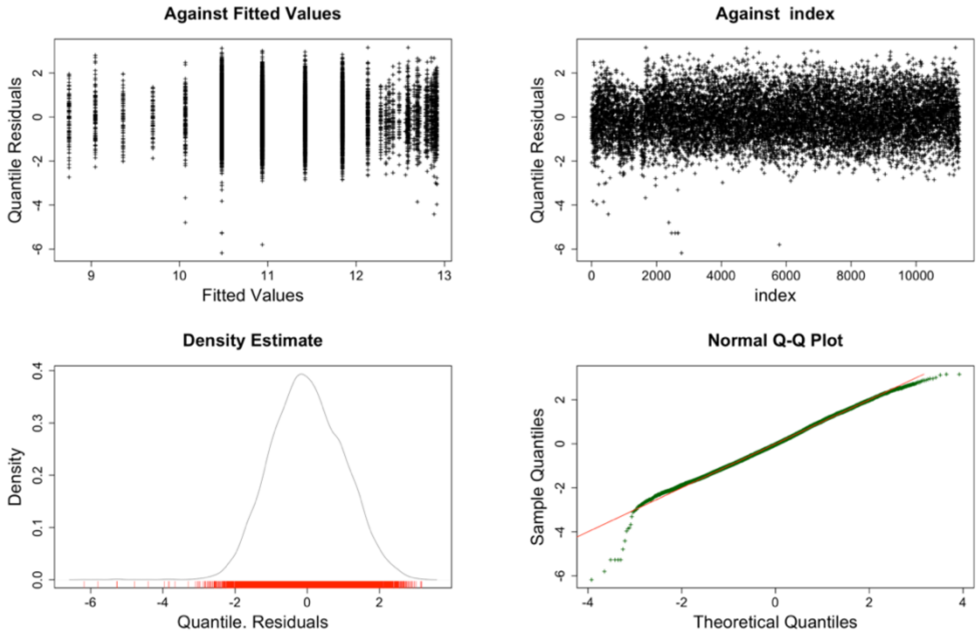

Figure S19 iTMT: Model fit diagnostic Q-Q plots, females. Visual inspection indicates that model residuals were normally distributed and supports the adequacy of the fitted distributions. iTMT = reproducible temporalis muscle thickness measurement.

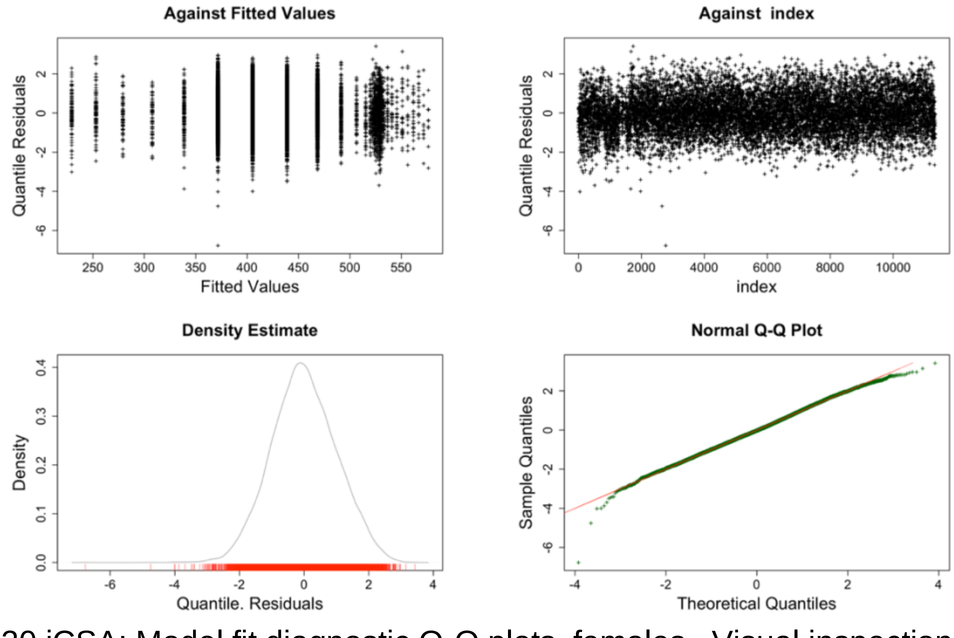

Figure S20 iCSA: Model fit diagnostic Q-Q plots, females. Visual inspection indicates that model residuals were normally distributed and supports the adequacy of the fitted distributions. iCSA = reproducible cross-sectional area measurement.

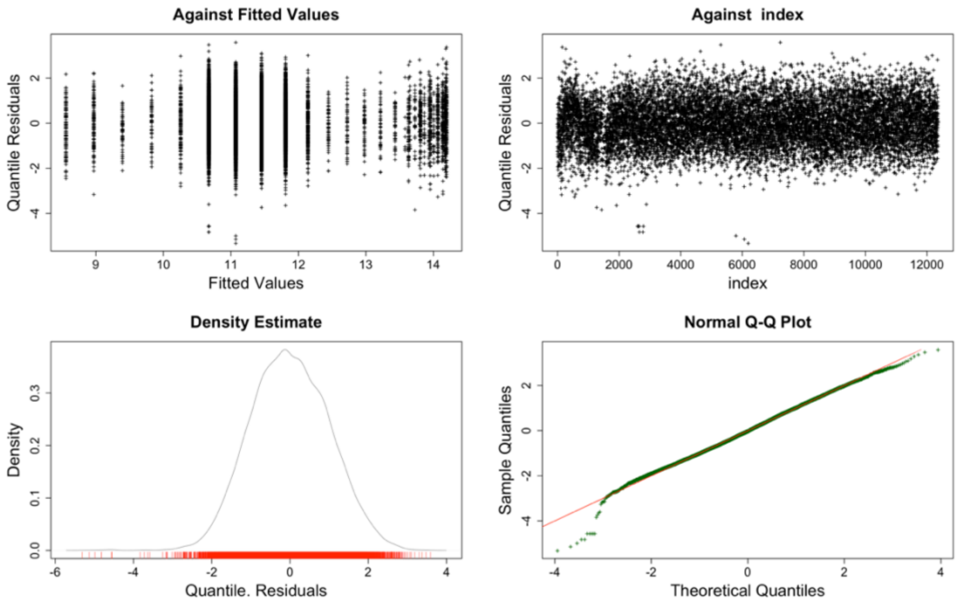

Figure S21 iTMT: Model fit diagnostic Q-Q plots, males. Visual inspection indicates that model residuals were normally distributed and supports the adequacy of the fitted distributions. iTMT = reproducible temporalis muscle thickness measurement.

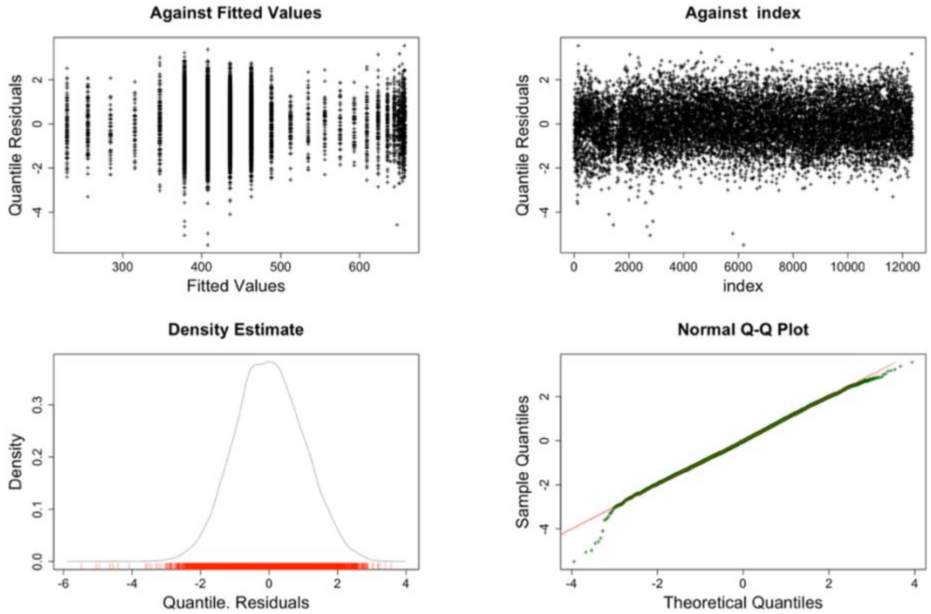

Figure S22 iCSA: Model fit diagnostic Q-Q plots, males. Visual inspection indicates that model residuals were normally distributed and supports the adequacy of the fitted distributions. iCSA = reproducible cross-sectional area measurement.

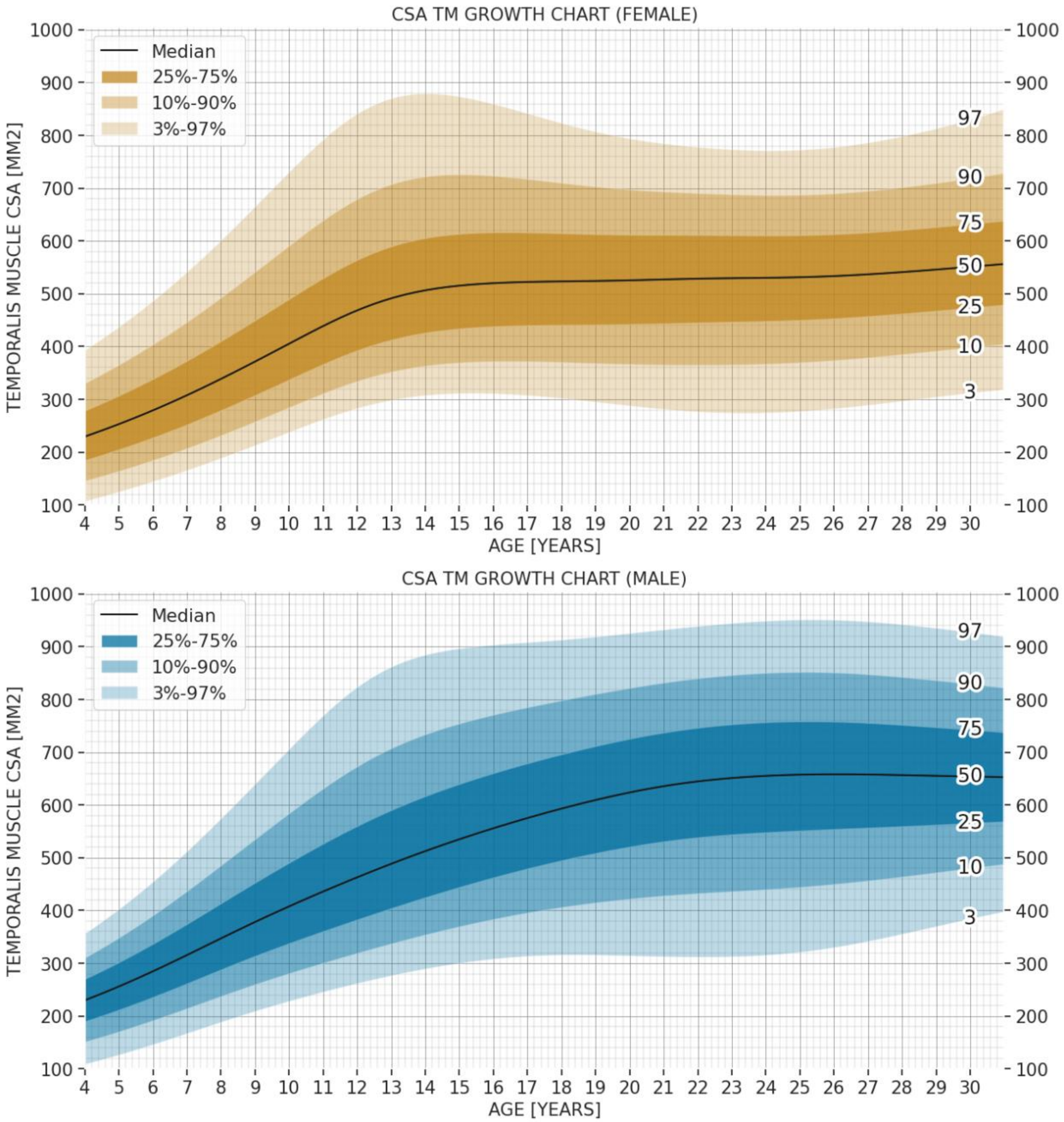

Figure S23 iCSA reference growth charts with percentiles for female (top) and male (bottom). These charts were developed based on applying iCSA to 23,396 T1w MRI scans for patients aged 4–30 years old and creating growth centile curves estimated using GAMLSS. Generalized Additive Models for Location Scale and Shape (GAMLSS). CSA = cross-sectional area; TM = temporalis muscle.

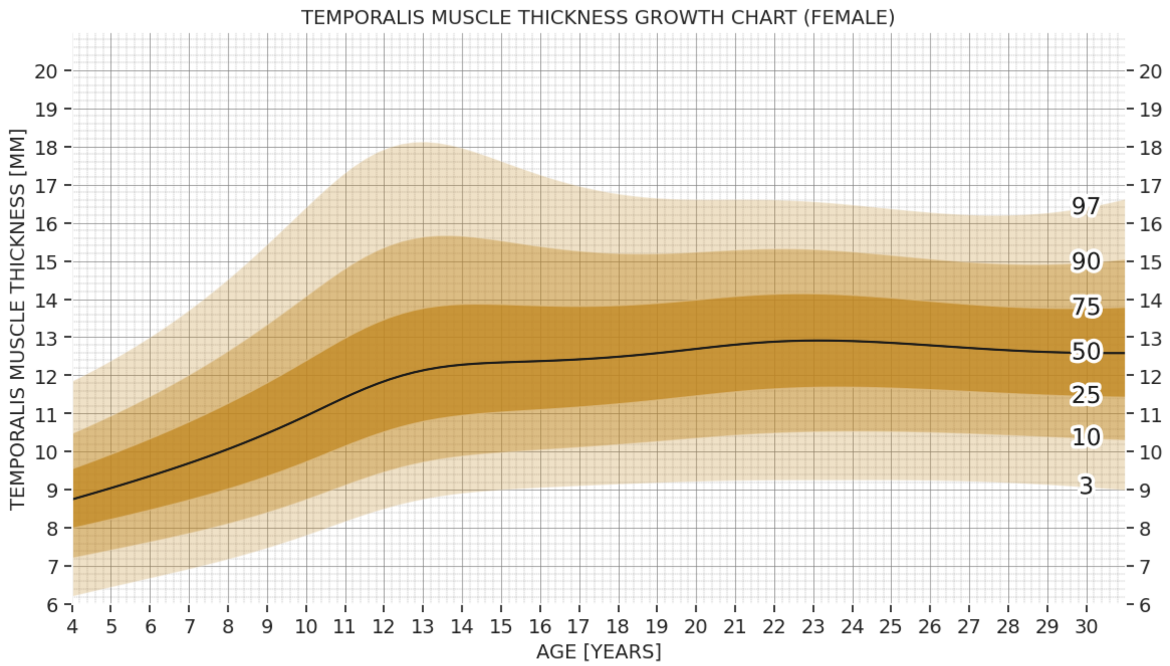

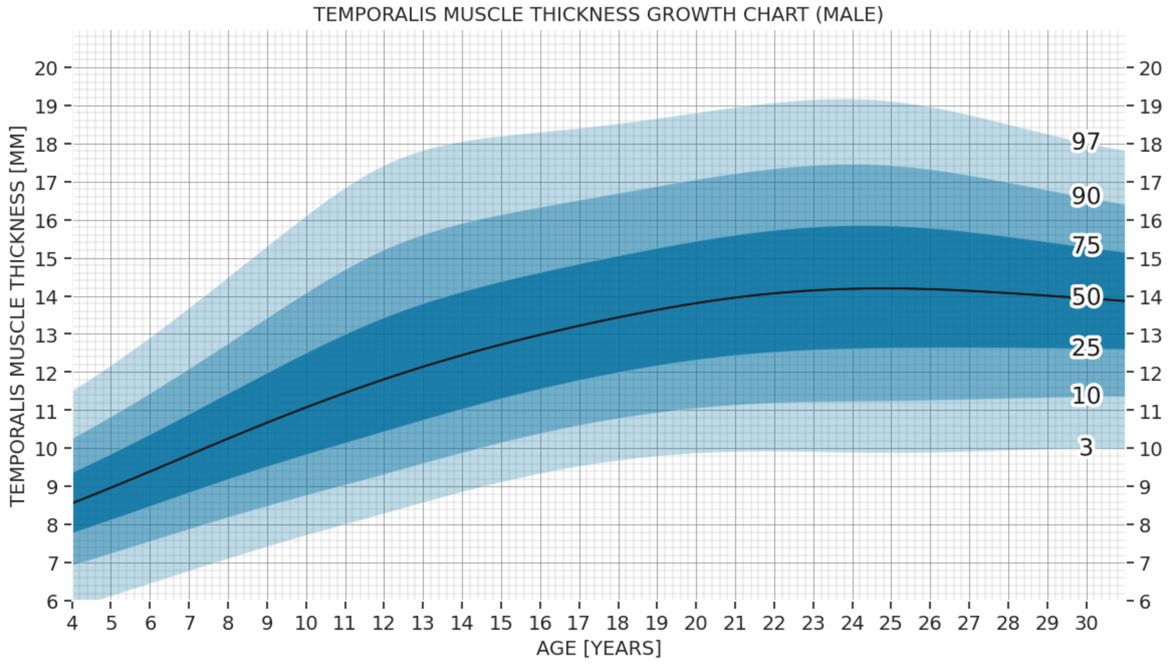

Figure S24 iTMT reference growth charts with percentiles for female (top) and male (bottom). These charts were developed based on applying iTMT to 23,396 T1w MRI scans for patients aged 4–30 years old and creating growth centile curves estimated using Generalized Additive Models for Location Scale and Shape (GAMLSS). CSA = cross-sectional area; TM = temporalis muscle.

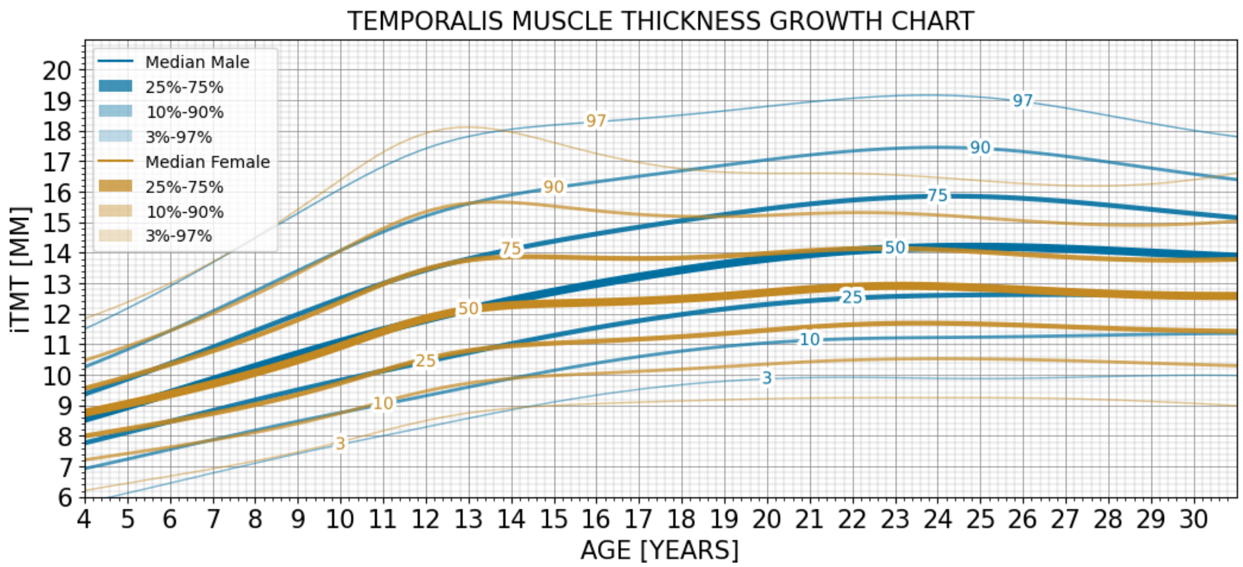

Figure S25 iTMT reference growth charts medians and quantile ranges overlayed for female and male. These charts were developed based on applying iCSA to 23,396 T1w MRI scans for patients aged 4–30 years old and creating growth centile curves estimated using GAMLSS. Generalized Additive Models for Location Scale and Shape (GAMLSS). CSA = cross-sectional area; TM = temporalis muscle.

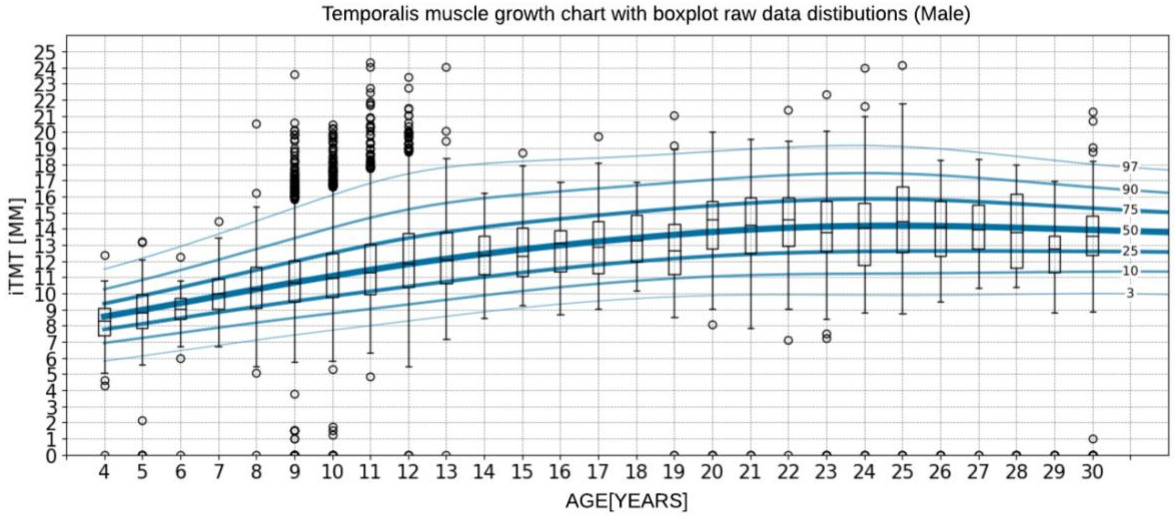

Figure S26 iTMT reference growth charts for male overlayed with raw data distributions(boxplots). These charts were developed based on applying iTMT to 23,396 T1w MRI scans for patients aged 4–30 years old. The box shows the first quartile to the third quartile (IQR). A line inside the box represents the median. Whiskers extend to the furthest value within 1.5 times the IQR from the box. Data points outside this are displayed individually as outliers.

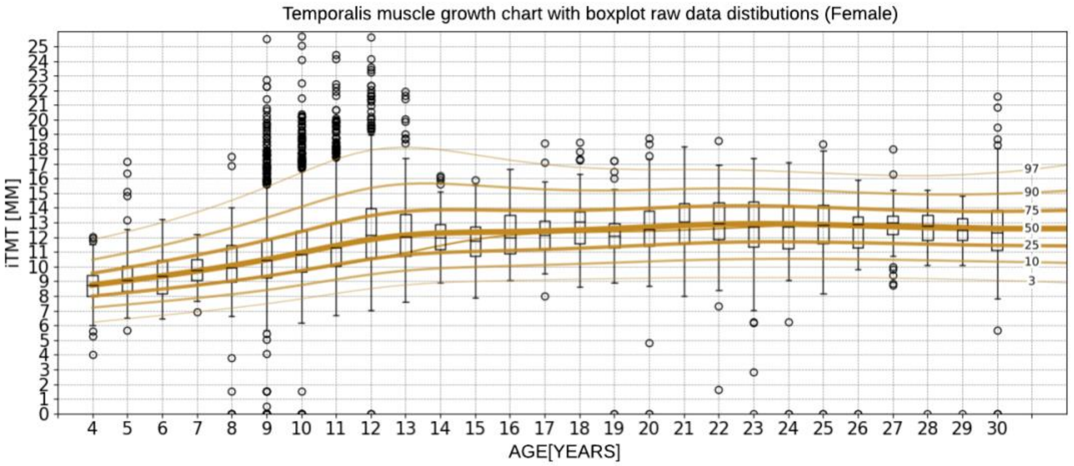

Figure S27 iTMT reference growth charts for female overlayed with raw data distributions(boxplots) These charts were developed based on applying iTMT to 23,396 T1w MRI scans for patients aged 4–30 years old. A line inside the box represents the median. Whiskers extend to the furthest value within 1.5 times the IQR from the box. Data points outside this are displayed individually as outliers.

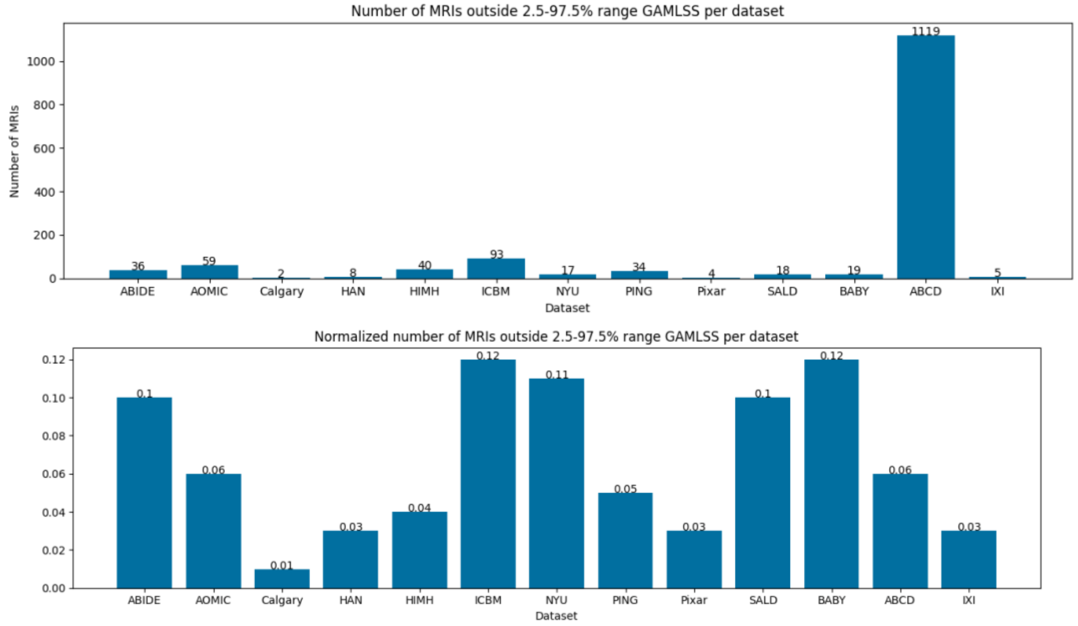

Figure S28 Frequency of outliers and dataset size. We calculate the percentage of TMT values that lie outside the range 2.5-97.5% (>3 standard deviations) within each dataset (23,396 T1w MRI). Top panel: the raw number of MRI outside centile range 2.5-97.5%, bottom panel: number of MRI outside centile range 2.5-97.5% normalized per

dataset size. An increase in study size results in a greater number of outliers.

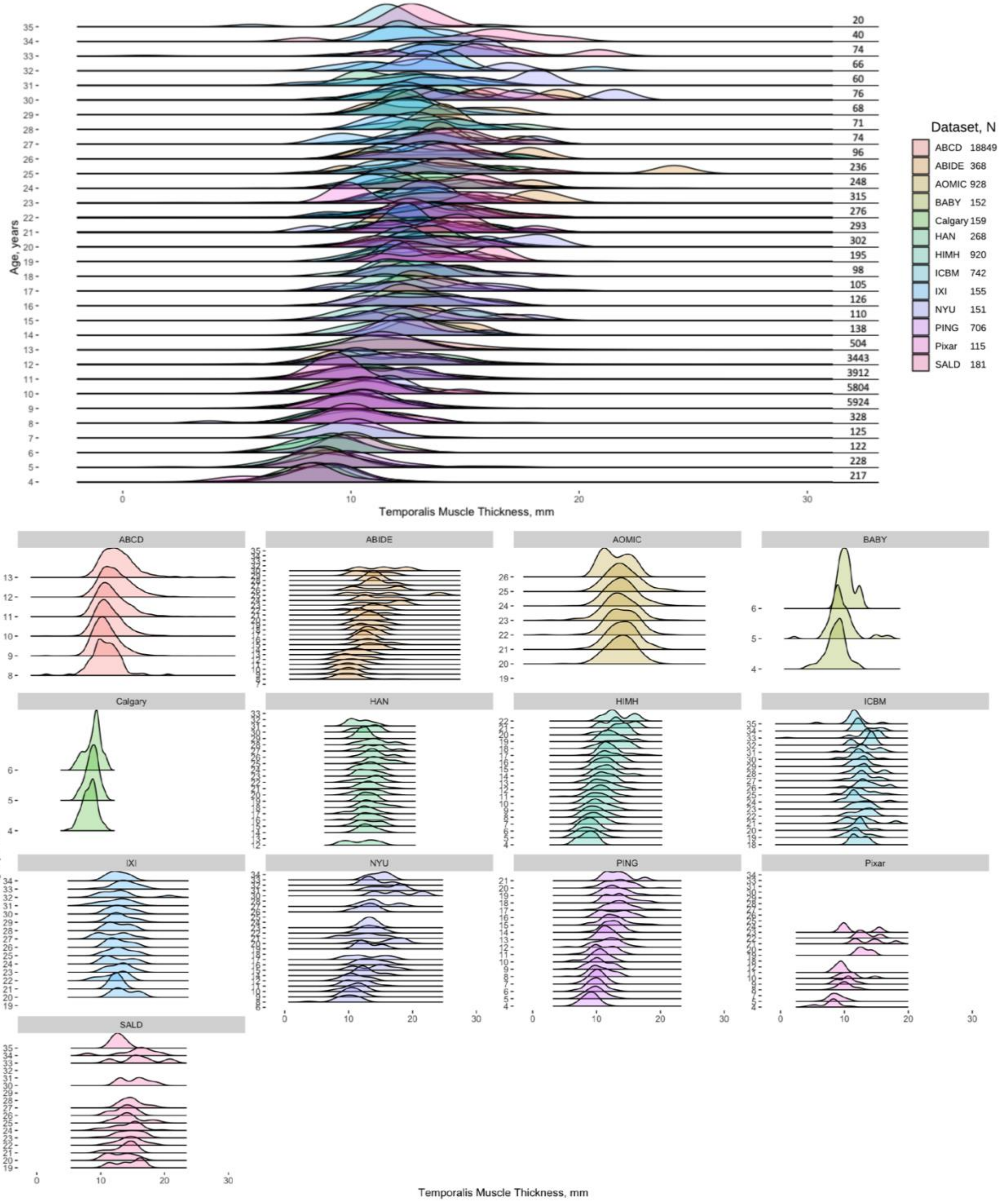

Figure S29 Ridgeline plots for dataset variability of temporalis muscle thickness(in mm) over ages 4-35. Top: all datasets together superimposed with several scans in each subset; bottom: ridgeline plot for each dataset.

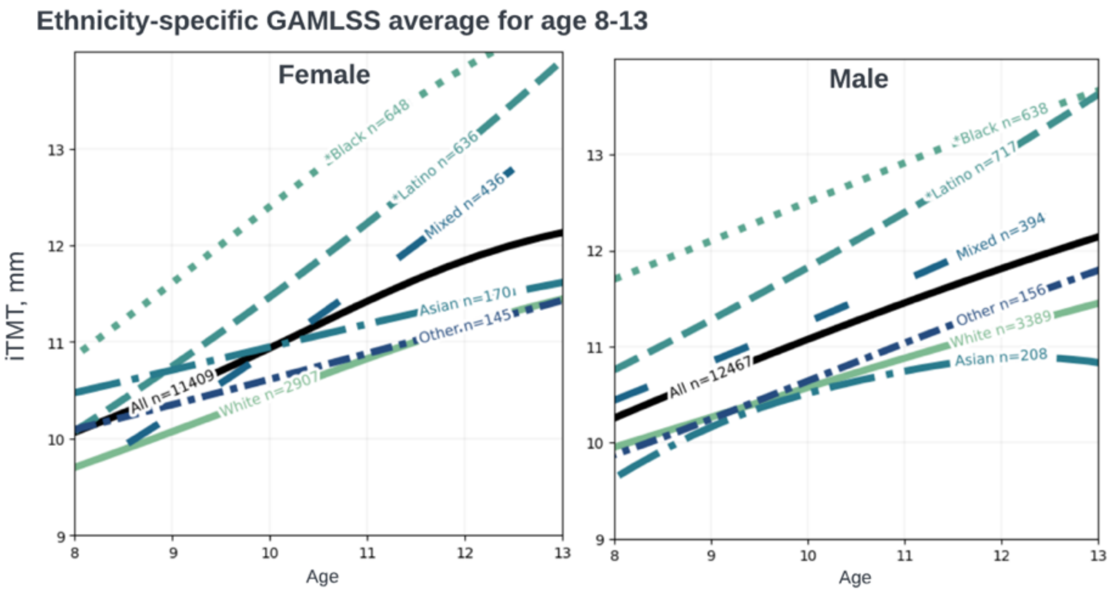

Figure S30 Ethnicity-specific median GAMLSS curves for female (left) and male(right) children aged 8-13 mean GAMLSS curve (50%). Blacks and Latinos had significantly higher iTMT scores (*p<0.05) than other ethnic groups. GAMLSS = Generalized Additive Models for Location Scale and Shape, iTMT= automated temporalis muscle thickness

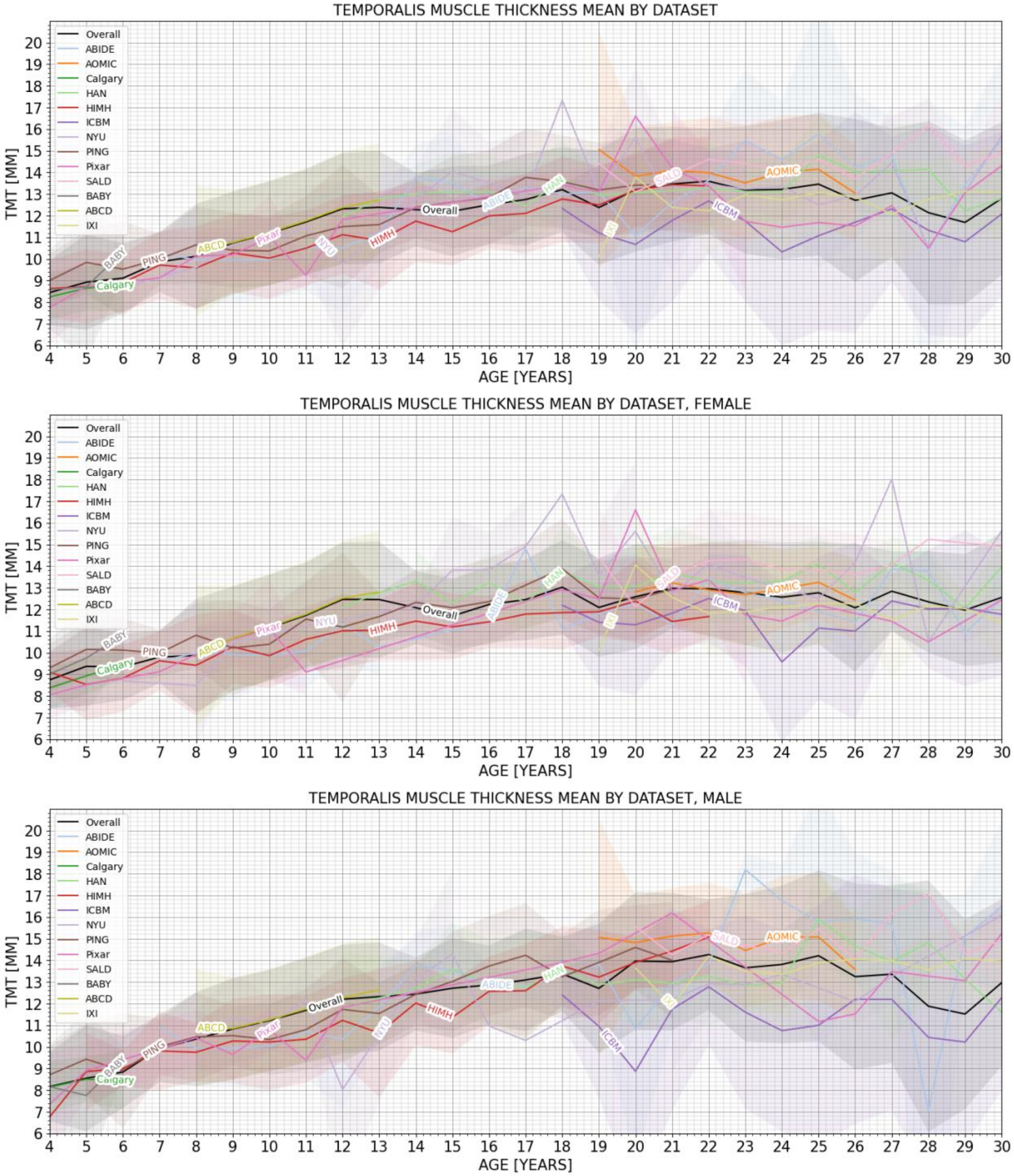

Figure S31 Standardized differences in mean temporalis muscle thickness(in mm) by age and data source(top: overall, middle: female only, bottom: male only). Plots reveal variations across data sources in standardized mean TMT within given developmental periods. Shaded areas correspond to 2.5%-97.5% Confidence intervals for each dataset.

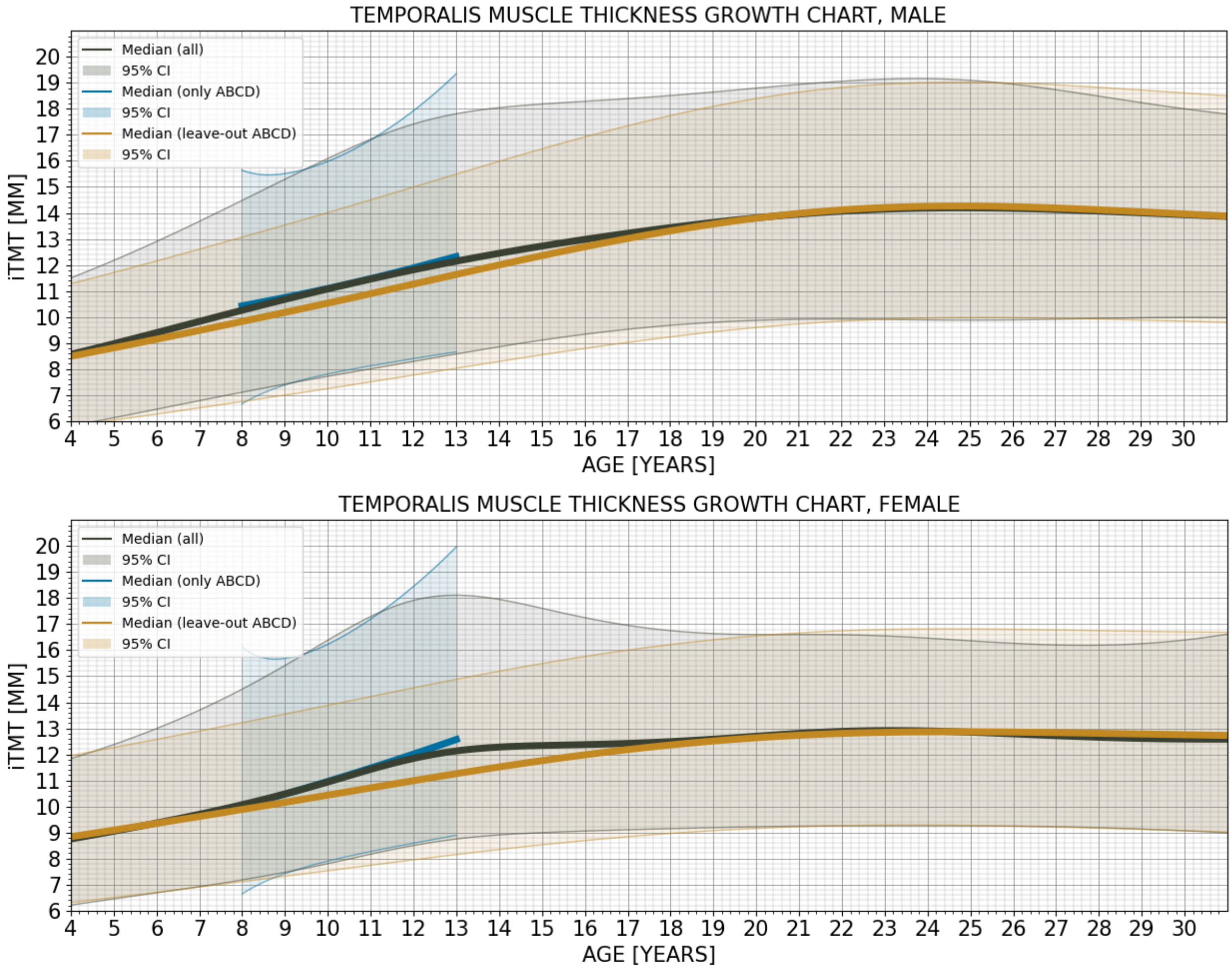

Figure S32 ABCD-specific fitted GAMLSS median curve with 95% confidence interval compared to the leave-out ABCD fitted GAMLSS median curve and overall GAMLSS curve.

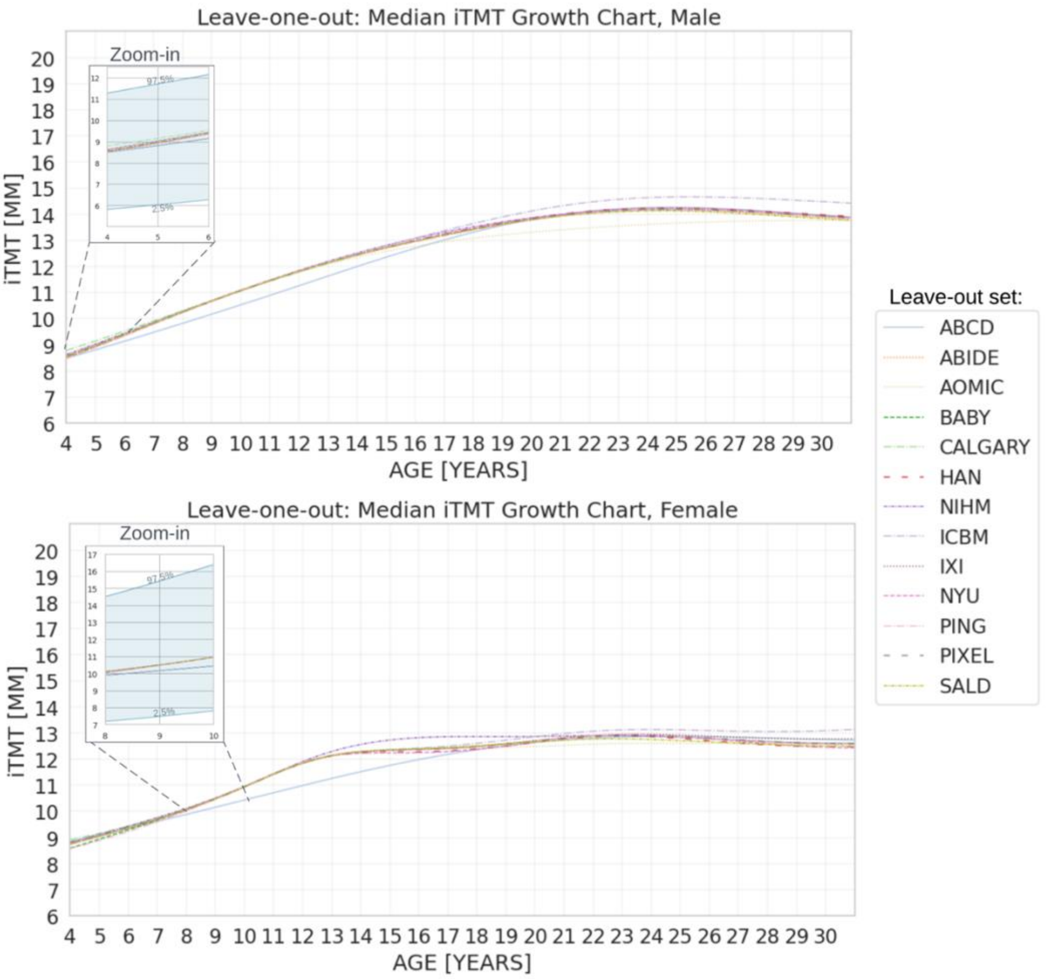

Figure S33 GAMLSS median curves fitted “leaving out” each dataset iteratively for male(top) and female(bottom).

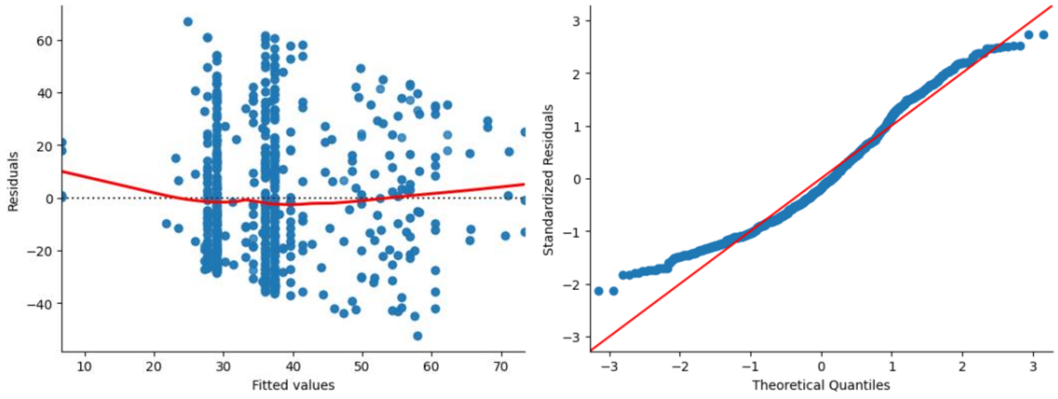

Figure S34 Left: Residuals vs Fitted plot and right: Normal Q-Q plot for multivariate model (Table S16). The residuals vs Fitted plot shows that there is non-linearity in the data. On the Q-Q plot, some points are far from the 45-degree-fitted line; however, most points lie close to the line, indicating that the residuals are mostly normally distributed.

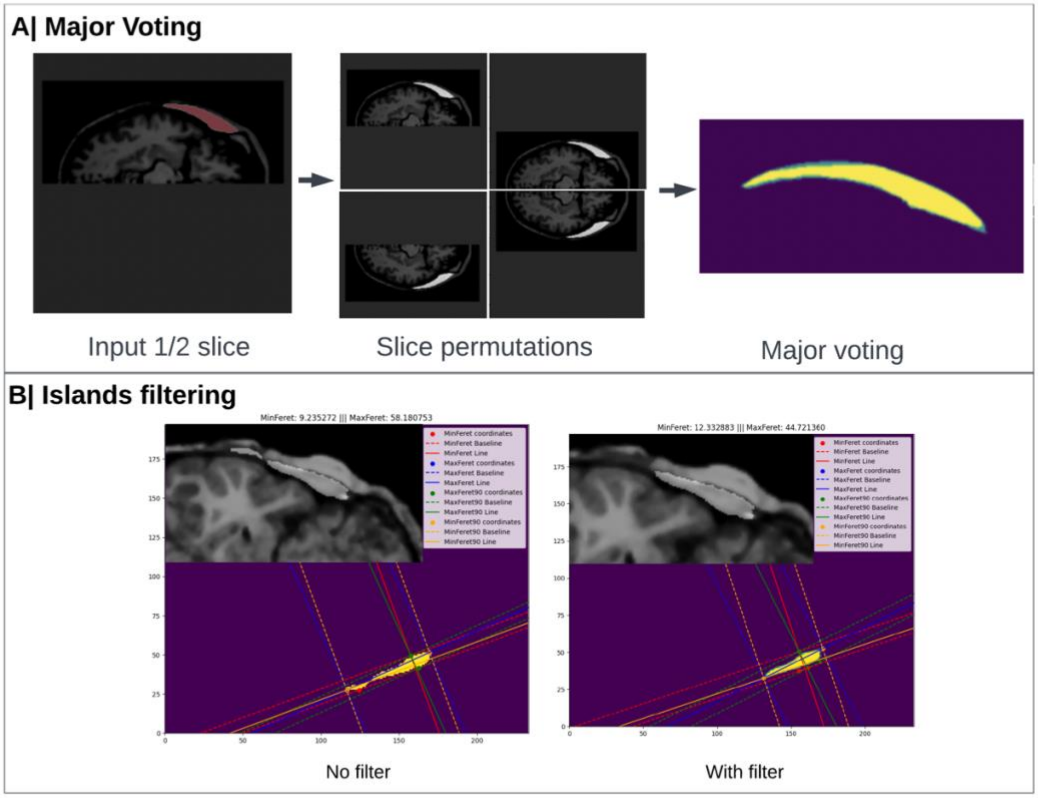

Figure S35 Overview of post-processing techniques for UNet segmentation: A) Major Voting (MV) – each temporalis muscle is permutated 4 times, and model predictions are combined via major voting (each pixel prediction is deemed as valid when it is present on 3 out of 4 predictions). B) Islands Filtering (IF) – volumes of predicted masks are computed, and only the bigger one is used for calculating the final thickness.

Supplementary Tables

Table S1 Agreement between two annotators measured over the cases where both annotators agreed with the registration pipeline with standard deviation(±). iTMT = automated temporalis muscle thickness measurement; CSA = cross-sectional area; MAE = mean absolute error; iCSA= automated cross-sectional area measurement

Cohort	Mean Age, years	N MRI	Registration Agreement	iTMT Slice Selection MAE, mm	Mean Dice	iCSA MAE, mm^2	iTMT MAE, mm
Healthy	13.2 IQR= [6-19]	50	4-disagreed; 46-agreed	3.06±2.5	0.84±0.04	53.3±36.9	1.01±0.75
Cancer	10.8 IQR= [7-15]	32	6-disagreed; 26-agreed	3.88±2.6	0.74±0.2	89.5±79.9	1.4±1.2

Table S2 One centile point changes in mm based on age and sex. On average, one iTMT centile point change translated to 0.296 mm (for females mean=0.149mm [95%CI: 0.124-0.173]; for males mean=0.176mm [95%CI: 0.15-0.2].

Age, years	Male, mm	Female, mm	Age, years	Male, mm	Female, mm	Age, years	Male, mm	Female, mm
4	0.12	0.08	13	0.24	0.22	22	0.21	0.17
5	0.13	0.11	14	0.08	0.07	23	0.22	0.17
6	0.12	0.07	15	0.09	0.08	24	0.24	0.17
7	0.08	0.05	16	0.08	0.08	25	0.18	0.18
8	0.21	0.17	17	0.11	0.1	26	0.18	0.16
9	0.27	0.25	18	0.07	0.1	27	0.18	0.09
10	0.03	0.26	19	0.21	0.17	28	0.18	0.15
11	0.19	0.2	20	0.2	0.19	29	0.17	0.15
12	0.23	0.26	21	0.2	0.1	30	0.21	0.22

Table S3 Impact of slice selection model error on the accuracy of CSA and temporalis muscle thickness (TMT) measurements on 47 test subject sets. iTMT = automated temporalis muscle thickness measurement; CSA = cross-sectional area; MAE = mean absolute error; iCSA= automated cross-sectional area measurement

**MAE slice**	**Avg MAE CSA, mm^2**	**Avg MAE TMT, mm**	**Avg % CSA**	**Avg % TMT**
+-2	13.44	0.42	1.77	1.85
+-3	17.9	0.54	2.22	2.36
+-4	20.65	0.63	2.57	2.73

Table S4 Subgroup cohort size analysis for iTMT grouped by BMI Levels. BMI normal reference tables were downloaded from the CDC[28]. iTMT=automated temporalis muscle thickness, BMI=body mass index (Dataset: ABCD [9]).

**iTMT Centiles Category**	**BMI Levels**	**Subgroup size**
<10	High	5
<10	Low	115
<10	Normal	1590
10-25	High	4
10-25	Low	148
10-25	Normal	2705
25-50	High	19
25-50	Low	133
25-50	Normal	4264
50-75	High	56
50-75	Low	54
50-75	Normal	3942
75-90	High	116
75-90	Low	12
75-90	Normal	2567
90>	High	389
90>	Low	4
90>	Normal	1590

Table S5 Summary of the fitted GAMLSS model (for males) for the TMT, showing the effective degrees of freedom (df) used in the model, criterion k, the BIC(AIC =log(4)), the GAIC (k = log(len(df)=log(12358)). BIC= Bayesian Information Criterion; GAIC= generalized Akaike information criterion, TMT = temporalis muscle thickness measurement.

Distribution	Degrees of freedom	GAIC	BIC
BCTo	18.5	54123.15	54123.15
BCPEo	38.1	54417.91	54111.50
BCCGo	51.6	54771.51	54356.68

Table S6 Summary of the fitted GAMLSS model (for females) for the TMT, showing the effective degrees of freedom used in the model, the BIC(AIC =log(4)), the GAIC (k = log(len(df)=log(11336)). BIC= Bayesian Information Criterion; GAIC= generalized Akaike information criterion, TMT = temporalis muscle thickness measurement.

Distribution	Degrees of freedom	GAIC	BIC
BCTo	20.4	49032.72	48870.61
BCPEo	20.9	49235.36	49068.86
BCCGo	18.9	49518.13	49367.75

Table S7 Dataset demographics for establishing healthy norms, excluding unknown ethnicity for N=10444 T1w MRI compared to nationwide USA percentages (Census [36]).

Race	Dataset, %	USA, %
White	60.2	58.9
Latino	12.9	16.6
Black	12.3	13.6
Asian	3.6	6.3
Mixed	7.9	3.0
Other	2.8	1.6

Table S8 Dataset demographics for establishing healthy normal reference charts: age mean, interquartile range(IQR), sex and number of MRIs.

Dataset	Age years, [min,max]	Age years, IQR	Age years, mean	Sex M/F	Number MRIs
ABCD	[8,13]	9-11	10	52.7%/47.3%	18849
ABIDE	[7,35]	9-15	11	65%/35%	336
AOMIC	[19,26]	21-24	22	48%/52%	924
Baby Connectome	[4,5]	4-5	4	50.9%/49.1%	151
Calgary	[4,6]	4-5	4	59.7%/40.3%	159
ICBM	[18,35]	22-30	25	54.5%/45.5%	433
IXI	[19,35]	24-31	28	51.6%/48.4%	155
NIMH	[4,22]	8-15	11	46.9%/53.1%	920
PING	[4,21]	8-17	12	51.3%/48.7%	705
Pixar	[4,34]	5-19	8	44.3%/55.7%	115
SALD	[19,35]	23-26	24	39.8%/60.2%	181
NYU2(CoRR)	[6,34]	11-22	15	37.5%/52.5%	150
Healthy adults	[18,35]	17-25	21	47.8%/52.2%	318

Table S9 Weight normal reference ranges. Source: [37]

Age	Males, pounds [min-max]	Males, kg [min-max]	Females, pounds [min-max]	Females, kg [min-max]
8	46-78	20.9-35.4	44-80	19.9-36.3
9	46-78	20.9-35.4	44-80	19.9-36.3
10	54-102	24.5-46.3	54-106	24.5-48.1
11	54-102	24.5-46.3	54-106	24.5-48.1
12	66-130	29.9-58.9	68-138	30.8-62.6
13	66-130	29.9-58.9	68-138	30.8-62.6

Table S10 Height normal reference ranges. Source: [37]

Age	Males, inches [min-max]	Males, cm [min-max]	Females, inches [min-max]	Females, cm [min-max]
8	47-54	119-137	47-54	119-137
9	47-54	119-137	47-54	119-137
10	50-59	127-150	50-59	127-150
11	50-59	127-150	50-59	127-150
12	54-64	137-163	55-64	140-163
13	54-64	137-163	55-64	140-163

Table S11 Dehydroepiandrosterone normal reference ranges. Source: [38]

Age	Males, ng/dL [min-max]	Females, ng/dL [min-max]
8	11-120	16-96
9	11-120	16-96
10	11-120	22-184
11	14-323	11-296
12	5.5 – 317.5	17-343
13	5.5 -312	37-738

Table S12 Testosterone (ERT) normal reference ranges. Source: [39]

Age	Males, ng/dL [min-max]	Females, ng/dL [min-max]
8	7 - 20	7 – 20
9	7 - 75	7 - 32
10	7 - 130	7 – 44
11	7 - 465	7 – 81.5
12	7 – 800	7-75
13	7 - 800	7-75

Table S13 Univariate and multivariate predictors of TMT centile (Dataset: ABCD[9]). OLS models, R squared for multivariate 0.14, F=15.13 (p=3.9e-32). This is a two-sided test; each coefficient was tested independently for statistical significance, no adjustments were made for multiple comparisons at this stage. OLS= Ordinary Least Squares, B-value= the coefficients of the independent variables and the constant term in the equation, TMT=temporalis muscle thickness.

	**UNIVARIATE ANALYSIS**		**MULTIVARIATE ANALYSIS**	
	**B (95% CI)**	**P value**	**B (95% CI)**	**P value**
**ETHNICITY**				
**WHITE/EUROPEAN**	1 [Reference]		1 [Reference]	
**LATINO**	15.6(6.6–24.7)	0.001	15.6(6.1–25.1)	0.000
**BLACK**	22.3(17.5–27.1)	0.000	20.8(15.5–26.05)	0.000
**MIXED**	20.1(13.6–26.6)	0.000	18.3(11.4–25.1)	0.000
**ASIAN**	13.4(-6.8–33.7)	0.194	21.8(-0.04–43.6)	0.05
**OTHER**	3.5(-5.7–12.6)	0.742	1.25(-8.2–10.7)	0.795
**CANNOT AFFORD FOOD IN THE LAST 12 MONTHS**				
**YES**	1 [Reference]		1 [Reference]	
**NO**	-9.5(-16.7–2.4)	0.009	2.9(-4.4–10.2)	0.438
**CHILD BORN IN USA**				
**YES**	1 [Reference]		1 [Reference]	
**NO**	-4.4(17.6–8.6)	0.5	-5.5(-18.9–8.0)	0.426
**FAMILY BORN IN USA**				
**YES**	1 [Reference]		1 [Reference]	
**NO**	-13.9(-27.8– -2e-3)	0.05	-22.3(-35.6 –8.9)	0.001
**HAS ANY TYPE OF INSURANCE**				
**YES**	1 [Reference]		1 [Reference]	
**NO**	10.5(6.6–14.4)	0.000	-1.8(-6.6–3.1)	0.474
**INCOME COMBINED**				
**<$50,000**	1 [Reference]		1 [Reference]	
**$50,000-$100,000**	-11.9(-16.8– -7.0)	0.000	-5.4(-11.0–0.24)	0.06
**>$100,000**	-15.2(-19.8– -10.6)	0.000	-4.1(-10–1.8)	0.176
**HIGHEST PARENT EDUCATION**				
**HIGH SCHOOL**	1 [Reference]		1 [Reference]	
**COLLEGE/ BACHELOR'S**	-20.8(-29.2– -12.5)	0.000	-12.9(-21.8– -4.2)	0.004
**MASTER'S, M.D., PH.D.**	-31.2(-39.8– -22.9)	0.000	-21.3(-30.5– -12.1)	0.000

Table S14 Comparison of post-processing techniques for UNet segmentation: Major Voting (MV) and Island Filtering (IF). The best result, the average MAE TMT 1.05 mm (Major Voting (MV) and Island Filtering (IF) combined), is highlighted in bold. CSA = cross-sectional area; MAE = mean absolute error; TMT = temporalis muscle thickness, Avg = average.

**Postprocessing method**	**Avg Dice**	**Median Dice**	**Avg % CSA**	**Avg % TMT**	**Avg MAE CSA**	**Avg MAE TMT**
No MV; no IF	0.835	0.848	13.4	8.6	107.04	1.99
MV; no IF	0.845	0.848	13.4	9.2	105.02	2.06
MV; IF	0.841	0.843	13.8	8.5	63.7	**1.05**

Supplementary References

1. Yushkevich, P. A. *et al.* User-guided 3D active contour segmentation of anatomical structures: significantly improved efficiency and reliability. *NeuroImage* **31**, 1116–1128 (2006).
2. Martini, M., Klausing, A., Lüchters, G., Heim, N. & Messing-Jünger, M. Head circumference - a useful single parameter for skull volume development in cranial growth analysis? *Head Face Med.* **14**, 3 (2018).
3. Nwt, M. Feret: A Python Module to calculate the Feret Diameter of Binary Images. (2023).
4. Esteban, O. *et al.* MRIQC: Advancing the automatic prediction of image quality in MRI from unseen sites. *PLOS ONE* **12**, e0184661 (2017).
5. Mortamet, B. *et al.* Automatic quality assessment in structural brain magnetic resonance imaging. *Magn. Reson. Med.* **62**, 365–372 (2009).
6. Stasinopoulos, D. M. & Rigby, R. A. Generalized Additive Models for Location Scale and Shape (GAMLSS) in R. *J. Stat. Softw.* **23**, 1–46 (2008).
7. Generalized additive models for location, scale and shape - Rigby - 2005 - Journal of the Royal Statistical Society: Series C (Applied Statistics) - Wiley Online Library. https://rss.onlinelibrary.wiley.com/doi/10.1111/j.1467-9876.2005.00510.x.
8. Evans, A. C. The NIH MRI study of normal brain development. *NeuroImage* **30**, 184–202 (2006).
9. Casey, B. J. *et al.* The Adolescent Brain Cognitive Development (ABCD) study: Imaging acquisition across 21 sites. *Dev. Cogn. Neurosci.* **32**, 43–54 (2018).
10. IXI Dataset – Brain Development. https://brain-development.org/ixi-dataset/.

11. Healthy Brain Network Data Portal. http://fcon_1000.projects.nitrc.org/indi/cmi_healthy_brain_network/.
12. MRI data of 3-12 year old children and adults during viewing of a short animated film. https://openfmri.org/dataset/ds000228/.
13. Howell, B. R. *et al.* The UNC/UMN Baby Connectome Project (BCP): An overview of the study design and protocol development. *NeuroImage* **185**, 891–905 (2019).
14. Children's Brain Tumor Network. https://cbtn.org/.
15. Gwet, K. Inter-rater reliability: dependency on trait prevalence and marginal homogeneity. *Stat. Methods Inter-Rater Reliab. Assess. Ser.* **2**, 9 (2002).
16. Di Martino, A. *et al.* Enhancing studies of the connectome in autism using the autism brain imaging data exchange II. *Sci. Data* **4**, 170010 (2017).
17. Snoek, L. *et al.* The Amsterdam Open MRI Collection, a set of multimodal MRI datasets for individual difference analyses. *Sci. Data* **8**, 85 (2021).
18. Reynolds, J. E., Long, X., Paniukov, D., Bagshawe, M. & Lebel, C. Calgary Preschool magnetic resonance imaging (MRI) dataset. *Data Brief* **29**, 105224 (2020).
19. Kötter, R. *et al.* A probabilistic atlas and reference system for the human brain: International Consortium for Brain Mapping (ICBM). *Philos. Trans. R. Soc. Lond. B. Biol. Sci.* **356**, 1293–1322 (2001).
20. Jernigan, T. L. *et al.* The Pediatric Imaging, Neurocognition, and Genetics (PING) Data Repository. *NeuroImage* **124**, 1149–1154 (2016).
21. Wei, D. *et al.* Structural and functional brain scans from the cross-sectional Southwest University adult lifespan dataset. *Sci. Data* **5**, 180134 (2018).

22. Zuo, X.-N. *et al.* An open science resource for establishing reliability and reproducibility in functional connectomics. *Sci. Data* **1**, 140049 (2014).
23. Nugent, A. C. *et al.* The NIMH intramural healthy volunteer dataset: A comprehensive MEG, MRI, and behavioral resource. *Sci. Data* **9**, 518 (2022).
24. Keator, D. B. *et al.* A National Human Neuroimaging Collaboratory Enabled by the Biomedical Informatics Research Network (BIRN). *IEEE Trans. Inf. Technol. Biomed.* **12**, 162–172 (2008).
25. The Alzheimer's disease neuroimaging initiative (ADNI): MRI methods - Jack - 2008 - Journal of Magnetic Resonance Imaging - Wiley Online Library. https://onlinelibrary.wiley.com/doi/full/10.1002/jmri.21049.
26. An open science resource for establishing reliability and reproducibility in functional connectomics | Scientific Data. https://www.nature.com/articles/sdata201449.
27. Pritschet, L. *et al.* Functional reorganization of brain networks across the human menstrual cycle. *NeuroImage* **220**, 117091 (2020).
28. World Health Organization. *WHO child growth standards : head circumference-for-age, arm circumference-for-age, triceps skinfold-for-age and subscapular skinfold-for-age : methods and development*. https://apps.who.int/iris/handle/10665/43706 (2007).
29. Dietary Recommendations for Healthy Children | American Heart Association. https://www.heart.org/en/healthy-living/healthy-eating/eat-smart/nutrition-basics/dietary-recommendations-for-healthy-children.
30. Tudor-Locke, C. *et al.* How many steps/day are enough? for children and adolescents. *Int. J. Behav. Nutr. Phys. Act.* **8**, 78 (2011).

31. High Cholesterol in Children and Teens. https://medlineplus.gov/highcholesterolinchildrenandteens.html.
32. Social Determinants of Health - Healthy People 2030 | health.gov. https://health.gov/healthypeople/priority-areas/social-determinants-health.
33. Seum, T., Meyrose, A.-K., Rabel, M., Schienkiewitz, A. & Ravens-Sieberer, U. Pathways of Parental Education on Children's and Adolescent's Body Mass Index: The Mediating Roles of Behavioral and Psychological Factors. *Front. Public Health* **10**, (2022).
34. Ogden, C. L. Prevalence of Obesity Among Youths by Household Income and Education Level of Head of Household — United States 2011–2014. *MMWR Morb. Mortal. Wkly. Rep.* **67**, (2018).
35. 3D Slicer image computing platform. *3D Slicer* https://slicer.org/.
36. U.S. Census Bureau QuickFacts: United States. https://www.census.gov/quickfacts/fact/table/US/PST045222.
37. Normal growth rates for kids | Children's Wisconsin. https://childrenswi.org/medical-care/adolescent-health-and-medicine/issues-and-concerns/adolescent-growth-and-development/normal-growth.
38. Dehydroepiandrosterone (DHEA): Reference Range, Interpretation, Collection and Panels. https://emedicine.medscape.com/article/2088870-overview?reg=1.
39. Testosterone, Total, Pediatric. https://www.healthcare.uiowa.edu/path_handbook/handbook/test97.html.
