# Supplementary material for: Automated temporalis muscle quantification and growth charts for children through adulthood
Source: Nat Commun. 2023 Nov 9;14:6863. doi: 10.1038/s41467-023-42501-1 (PMC10636102; doi:10.1038/s41467-023-42501-1)
Supplement: Supplementary file 3 — Reporting Summary [file 41467_2023_42501_MOESM3_ESM.pdf]

nature portfolio

Corresponding author(s): Dr Benjamin H. Kann

Last updated by author(s): Oct 18, 2023

Reporting Summary

Nature Portfolio wishes to improve the reproducibility of the work that we publish. This form provides structure for consistency and transparency in reporting. For further information on Nature Portfolio policies, see our Editorial Policies and the Editorial Policy Checklist.

Statistics

For all statistical analyses, confirm that the following items are present in the figure legend, table legend, main text, or Methods section.

n/a	Confirmed	
☐	☒	The exact sample size (*n*) for each experimental group/condition, given as a discrete number and unit of measurement
☐	☒	A statement on whether measurements were taken from distinct samples or whether the same sample was measured repeatedly
☐	☒	The statistical test(s) used AND whether they are one- or two-sided<br>*Only common tests should be described solely by name; describe more complex techniques in the Methods section.*
☐	☒	A description of all covariates tested
☐	☒	A description of any assumptions or corrections, such as tests of normality and adjustment for multiple comparisons
☐	☒	A full description of the statistical parameters including central tendency (e.g. means) or other basic estimates (e.g. regression coefficient) AND variation (e.g. standard deviation) or associated estimates of uncertainty (e.g. confidence intervals)
☐	☒	For null hypothesis testing, the test statistic (e.g. *F*, *t*, *r*) with confidence intervals, effect sizes, degrees of freedom and *P* value noted<br>*Give P values as exact values whenever suitable.*
☒	☐	For Bayesian analysis, information on the choice of priors and Markov chain Monte Carlo settings
☒	☐	For hierarchical and complex designs, identification of the appropriate level for tests and full reporting of outcomes
☐	☒	Estimates of effect sizes (e.g. Cohen's *d*, Pearson's *r*), indicating how they were calculated

Our web collection on statistics for biologists contains articles on many of the points above.

Software and code

Policy information about availability of computer code

Data collection	NDADownloadManager(v0.1.40), code is available at study repository https://doi.org/10.5281/zenodo.8428986
Data analysis	Python, Pydicom, SlicerElastix, Feret, Scipy. The model training and testing code is available in the study git repository: https://doi.org/10.5281/zenodo.8428986

For manuscripts utilizing custom algorithms or software that are central to the research but not yet described in published literature, software must be made available to editors and reviewers. We strongly encourage code deposition in a community repository (e.g. GitHub). See the Nature Portfolio guidelines for submitting code & software for further information.

Data

Policy information about availability of data

All manuscripts must include a data availability statement. This statement should provide the following information, where applicable:

- Accession codes, unique identifiers, or web links for publicly available datasets
- A description of any restrictions on data availability
- For clinical datasets or third party data, please ensure that the statement adheres to our policy

All data supporting the findings described in this manuscript are available in the article, in the Supplementary Information, and from the corresponding author upon request. Specifically, all public datasets used for this study can be found via the description and links in Supplementary Information A7. The BCH brain tumor dataset contains private hospital data that is controlled due to privacy concerns. Access to the derived dataset will be considered upon request to the corresponding author

(Benjamin H. Kann, M.D., email: Benjamin_Kann@dfci.harvard.edu, timeframe for response 2 weeks). All model parameters, weights, and details are provided in Supplementary Information A7. Source data are provided with this paper.

Research involving human participants, their data, or biological material

Policy information about studies with human participants or human data. See also policy information about sex, gender (identity/presentation), and sexual orientation and race, ethnicity and racism.

Reporting on sex and gender	In the study we used only the term sex as a biological attribute. We calculated sex-specific GAMLSS curves that explicitly estimate age-related variance, considering the known differences in development between males and females.
Reporting on race, ethnicity, or other socially relevant groupings	1. For the centile charts, we curated 23,852 MRIs from 13 datasets (90% United States; 52% male; 26% White, 6% Hispanic, 5% Black, 2% Asian, 4% Mixed, 1% Other; 56% Unknown).<br><br>2. To measure the generalizability of a proposed approach in children belonging to different ethnic groups, we fitted ethnic and gender-specific curves for ages 8-13 and focused solely on this age group due to limited demographic data for other age ranges. Our findings revealed that Black and Hispanic children displayed significantly higher median iTMT ($p<0.05$) compared to other ethnic groups, highlighting the importance of considering demographic factors when assessing iTMT (Supplementary Methods A11)
Population characteristics	1. We performed acceptability testing by two validators with an additional tie-breaker on 2,950 TM randomly selected segmentations (stratified by age/gender, age 4-35, mean age 18y) to ensure accurate predictions.<br>2. We applied iTMT to collected scans in the healthy cohort (n=23,852) and plotted sex-specific iTMT by age from 4 to 35 (Figure 3A). There was a median of 135 scans per age (IQR:81-299), with at least 60 scans for each year in the range of 4-30.<br>3. For the model training and testing, we annotated a dataset with patients aged 3 to 35 from 7 different imaging centers, stratified by age/gender, and performed data augmentation for each DL stage separately (327 MRI T1w unique patients, UNet training: 1120 TMs, DenseNet training: 45695 TMs, see details in Methodology section).
Recruitment	Since the dataset was aggregated from the 13+ primary studies, the recruitment are study specific. More details can be found in the Supplementary Material A7.
Ethics oversight	I confirm that all relevant ethical guidelines have been followed and that any necessary IRB and ethics committee approvals have been obtained. For the BCH dataset, the study was approved by the Institutional Review Board of Dana-Farber/Harvard Cancer Center, Protocol #13-055. We retrospectively collected information from the records of eligible patients under a waiver of consent. This study asks for a waiver of informed consent. The study involves no more than minimal risks to the participants. The rights and welfare of participants will not be adversely affected because their information will remain confidential and all treatment has already been rendered. It is not practicable to carry out this research without the waiver because we would like to examine older records. It is likely that a large percentage of those patients are deceased. It would not be feasible to find them or their next of kin. Without including the entire eligible population, subsequent analysis of the data would likely be skewed. There are no plans to share data with patients whose data is included in this study, as many patients received their treatment long ago and the data repository will have no impact on their care. The rest of datasets were anonymized, and not collected by the investigators, in which case the work is classified as non-human research.

Note that full information on the approval of the study protocol must also be provided in the manuscript.

Field-specific reporting

Please select the one below that is the best fit for your research. If you are not sure, read the appropriate sections before making your selection.

☒ Life sciences ☐ Behavioural & social sciences ☐ Ecological, evolutionary & environmental sciences

For a reference copy of the document with all sections, see nature.com/documents/nr-reporting-summary-flat.pdf

Life sciences study design

All studies must disclose on these points even when the disclosure is negative.

Sample size	We did not perform formal sample size calculations for this study. To obtain the most robust, representative dataset possible for the calculation of normal reference curves, we set out to collect as much data as possible, recognizing that construction of reliable GAMLSS model typically requires at least 100-150 observations and more complex models require at least 1000-1500 samples. Our final sample size was substantially more than this(n=23,852), and therefore sufficient for the calculation of reliable GAMLSS curves<br>1. For acceptability testing by two validators with an additional tie-breaker on 2,950 TM randomly selected segmentations, we determined sample size by the minimum available scans across gender and age strata.<br>2. For iTMT GAMLSS curve generation, we aimed to collect as much open source data as was available and feasible from open source datasets, though of uneven sizes. Open source datasets provide real-world variability that exists in the phenomenon of interest versus a highly controlled sample. In the healthy cohort (n=23,852) there was a median of 135 scans per age (IQR:81-299), with at least 60 scans for each year in the range of 4-30. While no firm minimum exists for GAMLSS, sample sizes in the hundreds are advisable for basic GAMLSS models, with larger samples needed for more complex distributions or models with more predictors.<br>3. For the model development and validation, we annotated a dataset with patients aged 3 to 35 from 7 different imaging centers, selected randomly and stratified by age/gender, and performed data augmentation for each deep learning stage separately (327 MRI T1w unique

patients, UNet training: 1120 TMs, DenseNet training: 45695 TMs, see details in Methodology section). Since manual data annotation of medical data type is costly and requires advanced degree, we aimed to collect at least 10 scans per each age group.

Data exclusions	The defacing protocols that deformed the temporalis muscle were excluded from the study. For the GAMLSS curves, "zero" iTMT model predictions were excluded from analysis.
Replication	To ensure the replication and reproducibility, we collected open source data from different institutions. We conduct intra-patient test to ensure the possibility of longitudinal tracking. All attempts at replication were successful.
Randomization	1. We performed acceptability testing by two validators with an additional tie-breaker on 2,950 TM randomly selected segmentations (stratified by age/gender, age 4-35, mean age 18y) .<br>2. For the model training and testing, we annotated a dataset with patients randomly drawn from the cohort aged 3 to 35 from 7 different imaging centers, stratified by age/gender.
Blinding	All readers were blinded to the demographic and clinical characteristics of patients.

Reporting for specific materials, systems and methods

We require information from authors about some types of materials, experimental systems and methods used in many studies. Here, indicate whether each material, system or method listed is relevant to your study. If you are not sure if a list item applies to your research, read the appropriate section before selecting a response.

Materials & experimental systems

n/a	Involved in the study
☒	☐ Antibodies
☒	☐ Eukaryotic cell lines
☒	☐ Palaeontology and archaeology
☒	☐ Animals and other organisms
☐	☒ Clinical data
☒	☐ Dual use research of concern
☒	☐ Plants

Methods

n/a	Involved in the study
☒	☐ ChIP-seq
☒	☐ Flow cytometry
☐	☒ MRI-based neuroimaging

Clinical data

Policy information about clinical studies
All manuscripts should comply with the ICMJE guidelines for publication of clinical research and a completed CONSORT checklist must be included with all submissions.

Clinical trial registration	For the BCH dataset, the study was approved by the Institutional Review Board of Dana-Farber/Harvard Cancer Center, Protocol #13-055 as secondary use research. The rest of datasets are secondary use data that are from publicly accessible repositories, anonymised, and were not originally collected by the investigators, in which case the work is classified as non-human subject research. All data used in this retrospective study was acquired prior to study initiation.
Study protocol	Institutional Review Board of Dana-Farber/Harvard Cancer Center, Protocol #13-055
Data collection	Pediatric imaging data is obtained through the Boston Children's Hospital system. To create the data repository, all required raw data is downloaded onto a limited-access shared drive within the Partners environment. Only study staff will have access to this patient information for the purposes of data collection. The data collection presents a minimal risk to the patients.
Outcomes	To examine the role of AI-derived features for clinical validity, by performing a quantitative analysis of medical image data through automatic or semi-automatic software of a given imaging modality to assess if this can provide more and better information than would be obtained by a typical reading of that image by a physician.

Magnetic resonance imaging

Experimental design

Design type	The dataset was aggregated from 13+ primary studies, see Supplementary Material A7 for the MRI scanning protocols and details on the demographics data
Design specifications	Supplementary Material A7
Behavioral performance measures	None

Acquisition

Imaging type(s)	Structural
Field strength	Supplementary Material A7
Sequence & imaging parameters	Supplementary Material A7
Area of acquisition	Head

Diffusion MRI ☐ Used ☒ Not used

Preprocessing

Preprocessing software	Python 3.9, pydicom, nibabel, simpleITK. The overall pipeline of the proposed method is shown in Figure 1C. First, we pre-processed MRI scans by applying registration and image normalization methods. Scans that were downloaded in native DICOM format were converted to NifTI via Python Pydicom package. Next, scans were co-registered to MRI age-dependent T1- weighted asymmetric brain atlases, generated from the NIH-funded MRI Study of Normal Brain Development (hereafter, NIHPD, for NIH pediatric database) with rigid registration using SlicerElastix(Elastix generic rigid preset).
Normalization	Denoising(kenel size=3), intensity rescaling(percentile range=[0.5,99.5], number of bins=256), histogram equalization(number of bins=256), z-normalization, otsu-filtering(nbins=6). Details are described in Methodology section and can be found in the open source code repository.
Normalization template	Scans were co-registered to MRI age-dependent T1- weighted asymmetric brain atlases82, generated from the NIH-funded MRI Study of Normal Brain Development (hereafter, NIHPD, for NIH pediatric database) with rigid registration using SlicerElastix (Elastix generic rigid preset).
Noise and artifact removal	See above
Volume censoring	During the curation of the open-source datasets, we needed to eliminate multiple good studies due to anonymization techniques performed on the MRI scans that deformed the temporalis muscle.

Statistical modeling & inference

Model type and settings	Predictive modeling, see Methodology section
Effect(s) tested	The temporalis muscle thickness and cross-sectional area were modeled using GAMLSS curves and tested by gender/age.

Specify type of analysis: ☐ Whole brain ☒ ROI-based ☐ Both

Anatomical location(s)	To measure TMT and CSA, we use python minimum Feret diameter implementation . Measurements were conducted at each side at the level of the orbital roof (cranio–caudal landmark) and the lateral sulcus (anterior-posterior landmark) perpendicular to the long axis of the temporal muscle. We followed the previous study by Steindl et al., measuring TMT values on both sides and dividing them by two to calculate the mean TMT values for each patient and reduce dental- or oral-related muscle changes.
Statistic type for inference (See Eklund et al. 2016)	No fMRI used
Correction	No fMRI used

Models & analysis

n/a	Involved in the study
☒	☐ Functional and/or effective connectivity
☒	☐ Graph analysis
☐	☒ Multivariate modeling or predictive analysis

Multivariate modeling and predictive analysis	1. DenseNet model.We trained the model using Adam optimizer for 30 epochs with batch size 64 and mean squared error (MSE) loss with an initial learning rate 1e-4. We set up the learning rate scheduler to reduce on a plateau with starting learning rate=5e-4 and use 1x Nvidia A6000 for training with TensorFlow v.2.10, Python v.3.9<br>2. UNet model. we trained 2D UNet for 30 epochs with batch size 4 with an initial learning rate 5e-4 and the same strategy for the learning rate decreased as described earlier. We upscaled images into 512x512 and use five downsampling/upsampling modules. We add data augmentations, including 10 deg rotations, width and height shifts, horizontal flips, and zooming. We use Focal Tversky Loss proposed by Nabila Abraham et al.<br>3. To measure the association of iTMT with social and demographic factors in children, we fitted univariable and multivariable linear regressions for iTMT percentile as a target variable, including total household

income, highest parent education, insurance, country of birth for child and family(inside or outside USA), food insecurity and race/ethnicity (total of 1227 records, Multiple R-squared: 0.14, F=15.13 (p=3.9e-32). We conducted this analysis using the ABCD dataset, which captures rich metadata, and thus analyses were limited to the 8-13-year-old United States group, which may limit generalizability to other countries and age groups.
